# Different evolutionary pathways underlie the morphology of wrist bones in hominoids

**DOI:** 10.1186/1471-2148-13-229

**Published:** 2013-10-23

**Authors:** Tracy L Kivell, Anna P Barros, Jeroen B Smaers

**Affiliations:** 1 School of Anthropology and Conservation, University of Kent, Canterbury, UK; 2 Department of Human Evolution, Max Planck Institute for Evolutionary Anthropology, Leipzig, Germany; 3 Department of Anthropology, University College London, London, UK; 4 Department of Anthropology, Stony Brook University, Stony Brook, USA; 5 Department of Genetics, Evolution and Environment, University College London, London, UK

**Keywords:** Carpal, Functional morphology, Locomotion, Phylogeny, Variable rates estimation

## Abstract

**Background:**

The hominoid wrist has been a focus of numerous morphological analyses that aim to better understand long-standing questions about the evolution of human and hominoid hand use. However, these same analyses also suggest various scenarios of complex and mosaic patterns of morphological evolution within the wrist and potentially multiple instances of homoplasy that would benefit from require formal analysis within a phylogenetic context.

We identify morphological features that principally characterize primate – and, in particular, hominoid (apes, including humans) - wrist evolution and reveal the rate, process and evolutionary timing of patterns of morphological change on individual branches of the primate tree of life. Linear morphological variables of five wrist bones – the scaphoid, lunate, triquetrum, capitate and hamate – are analyzed in a diverse sample of extant hominoids (12 species, 332 specimens), Old World (8 species, 43 specimens) and New World (4 species, 26 specimens) monkeys, fossil Miocene apes (8 species, 20 specimens) and Plio-Pleistocene hominins (8 species, 18 specimens).

**Result:**

Results reveal a combination of parallel and synapomorphic morphology within haplorrhines, and especially within hominoids, across individual wrist bones. Similar morphology of some wrist bones reflects locomotor behaviour shared between clades (scaphoid, triquetrum and capitate) while others (lunate and hamate) indicate clade-specific synapomorphic morphology. Overall, hominoids show increased variation in wrist bone morphology compared with other primate clades, supporting previous analyses, and demonstrate several occurrences of parallel evolution, particularly between orangutans and hylobatids, and among hominines (extant African apes, humans and fossil hominins).

**Conclusions:**

Our analyses indicate that different evolutionary processes can underlie the evolution of a single anatomical unit (the wrist) to produce diversity in functional and morphological adaptations across individual wrist bones. These results exemplify a degree of evolutionary and functional independence across different wrist bones, the potential evolvability of skeletal morphology, and help to contextualize the postcranial mosaicism observed in the hominin fossil record.

## Background

A detailed understanding of the evolutionary patterns and processes that underlie skeletal morphology are crucial to explaining present-day diversity and interpreting the fossil record. The process of morphological evolution has long been viewed as one of a clear form-function relationship involving gradual adaptation to specific biota [1]. However, fossil evidence of mosaic and/or convergent evolution within or across different morphological modules [2-7] demonstrates that the evolutionary process is more complex. Comparative morphological analyses within a phylogenetic framework help to unravel the complex patterns of skeletal morphological evolution [8-10] and are essential for 1) assessing evolutionary trends [10,11], 2) inferring changes in rates of evolution [12], and 3) ascertaining whether observed similarities result from common descent or are acquired independently [8,13].

Order Primates is an ideal clade to further our understanding of skeletal morphological evolution within a phylogenetic context. Primates are characterised by a diverse range of locomotor adaptations, including behaviours not seen in other extant mammals, such as vertical clinging and leaping, ricochetal brachiation, and striding, straight-leg bipedalism. Within primates, the functional morphology of wrist and hand is of particular interest because an increased reliance on the hindlimbs to power locomotion [14] promotes a decoupling of hind- and forelimb functions, leaving the forelimb free to be used more for movements of guidance, grasping and manipulation [15].

The hominoid wrist has received special attention because of its implications for human evolutionary history, including the mode of locomotion from which bipedalism emerged [16-20], the degree of arboreality in early hominins [4,21-23], and the evolution of human dexterity [22,24-27]. In particular, features shared between the human and African ape wrist have been interpreted as evidence that human bipedalism evolved from a knuckle-walking ancestor [8,17,28,29], while others consider these features to have evolved in parallel in African apes and their presence in humans to be largely the result of a close phylogenetic relationship to *Pan*[18,20,30]. The interpretation of *Ardipithecus ramidus* as distinctly unlike extant hominoids prompted the researchers to propose that all knuckle-walking, climbing and suspensory features, including those of the wrist and hand, shared by extant hominoids evolved in parallel [31]. Furthermore, fossil hominoids and hominins demonstrate a high level of postcranial mosaicism - the concept that certain morphological features may undergo evolutionary change at different rates than other morphological features in a lineage [32] [e.g. *Sivapithecus*[33,34]; *Ar. ramidus*[31], *Australopithecus sediba*[3,35,36], *Au. afarensis*[4]] -complicating the interpretation of the evolutionary pathways of particular morphologies.

Part of the debate over the processes that characterize the morphological evolution of the hominoid wrist, and skeletal morphology in general, may result from the methods that have been used to infer its evolutionary history. Although homoplasies can only be identified within a phylogenetic context, many of the hypotheses of parallel evolution within hominoids are largely post hoc interpretations without a formal analytical consideration of phylogeny. Studies utilizing morphologically-based phylogenetic inferences to quantify rates of evolution [8,37-42] assume a clear association between morphological and evolutionary diversity that is highly problematic in cases of mosaic evolution. Previous research on primate hard and soft tissue anatomy, for example, has shown varying degrees of congruence between the morphology-based phenetic trees and molecular-based phylogenies [8,43,44].

**Figure 1:**
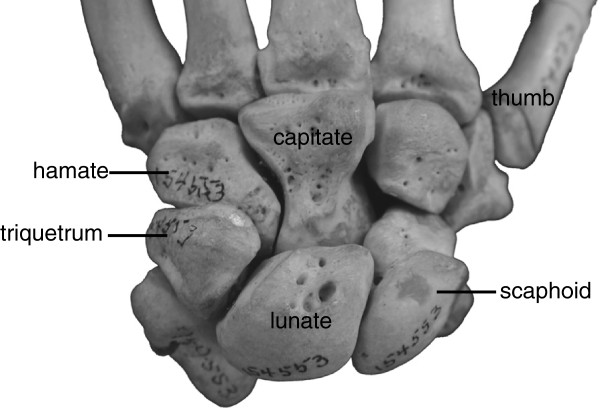
Gorilla wrist in dorsal view.

To understand the patterns of phenotypic change that occur in relation to genotypic diversification, phenotypic data can be mapped onto an independently-estimated, molecular-based phylogenetic tree [12,45]. This approach highlights processes of phenotypic change occurring across individual branches of a phylogeny and therefore has the potential to identify processes such as convergence and mosaic evolution within the skeleton. Here, we apply this phylogenetically-integrated approach to quantify evolutionary changes in primate wrist morphology (38 linear variables on five wrist bones, Figure 1 and Additional files 1 and 2) over 47 million years of evolution and across 24 extant primate species (401 specimens; Table 1) and 16 fossil primate taxa (38 specimens; Table 2) of varying locomotor behaviours. This approach moves away from direct species comparisons by (1) utilizing independently-estimated (molecular) phylogenies to identify which morphological signals dominate the evolution of an anatomical module (i.c., the wrist) and (2) inferring the timing and rate of evolutionary changes along individual lineages. By quantifying evolutionary changes along individual branches of the tree of life, our approach allows robust inferences of instances of independent evolution and provides a useful framework to help interpret fossil morphology, especially in cases where taxonomy is uncertain. Here, we explore the evolutionary pathways that underlie extant and fossil hominoid wrist morphology within a phylogenetic context to test previously proposed hypotheses of independent evolution and to explore the potential for mosaic evolution within this key morphological area.

**Table 1:** Extant primate sample used in this study

**Group**	**Taxon**	***N***	**Body mass (kg) range F-M**^**a**^	**Locomotor behaviours**
NWM	*Lagothrix lagotricha*	7	7.0-9.3	arboreal quadruped, with climbing and orthograde clambering^b^
	*Ateles* sp.	4	7.3-8.9	torso-orthograde suspensory; semi-brachiator^c^
	*Alouatta* sp.	13	5.4-7.2	arboreal quadruped, with climbing and orthograde clambering
Hominoids	*Pongo pygmaeus*	25	35.8-78.5	torso-orthograde suspensory^d^
	*Pongo abelii*	9	35.6-77.9	torso-orthograde suspensory
	*Pan troglodytes verus*	14	41.6-46.3	knuckle-walker (arboreal and terrestrial) and climbing^e^
	*Pan troglodytes troglodytes*	25	45.8-59.7	knuckle-walker (arboreal and terrestrial) and climbing
	*Pan troglodytes schweinfurthii*	6	33.7-42.7	knuckle-walker and climbing (proportion of arboreality varies from 33-68%)
	*Pan paniscus*	19	33.2-45.0	knuckle-walker (arboreal and terrestrial) and climbing, considered more arboreal than *P. troglodytes*^f^
	*Homo sapiens*	146	54.4-62.2	terrestrial biped
	*Gorilla gorilla gorilla*	40	71.5-170.4	terrestrial knuckle-walker (assumed to be less terrestrial than *G.b.beringei*)^g^
	*Gorilla beringei graueri*	7	71.0-175.2	terrestrial knuckle-walker (assumed to be less terrestrial than *G.b.beringei*)
	*Gorilla beringei beringei*	9	97.5-162.5	terrestrial knuckle-walker (most terrestrial of all *Gorilla*, 93-98% terrestrial)
	*Symphalangus syndactylus*	5	10.7-11.9	brachiator^h^
	*Hylobates lar*	27	5.4-5.9	brachiator
OWM	*Presbytis* sp.	2	5.6-6.8	arboreal quadruped, capable of leaping and forelimb suspension^i^
	*Macaca mulatta*	16	8.8-11.0	semi-terrestrial quadruped^j^
	*Macaca fascicularis*	7	3.6-5.4	arboreal quadruped^k^
	*Papio Anubis*	6	13.3-25.1	terrestrial quadruped^l^
	*Theropithecus gelada*	5	11.7-19.0	terrestrial quadruped
	*Chlorocebus aethiops*	4	3.0-5.5	semi-terrestrial quadruped
	*Erythrocebus patas*	3	6.5-12.4	terrestrial quadruped
	*Cercopithecus mitis*	12	3.9-5.9	arboreal quadruped

Although we recognize that most primate taxa engage in a wide range of locomotor and postural behaviours, our summary here is only a brief description of the most frequent locomotor behaviour and environment.

^a^ Smith and Jungers (1997).

^b^ Describes a variety of locomotor behaviors, including quadrupedalism, climbing, and orthograde clambering, in an arboreal context (Cant et al., 2001, 2003).

^c^ Describes both torso-orthograde clambering and brachiation, which make up 50% of arboreal locomotion (Cant et al., 2001, 2003).

^d^ Describes both torso-orthograde clambering and brachiation which make up 35-60% of arboreal locomotion (Cant, 1987; Thorpe and Crompton, 2006).

^e^ Describes both terrestrial knuckle-walking – the primary mode of locomotion – in addition to various arboreal locomotor behaviours, including knuckle-walking, vertical climbing, clambering and suspension (Hunt 1991; Doran, 1996).

^f^ Doran (1992, 1993).

^g^ Hunt (1992) and Doran (1996).

^h^ Hunt (1991).

^i^ Fleagle (1977).

^j^ Describes OWM that engage in roughly equal time in both terrestrial and arboreal environments (Rose, 1979; Wells and Turnquist, 2001).

^k^ Describes OWM that engage primarily in quadrupedalism in an arboreal environment (Cant, 1988; Gebo and Chapman, 1995).

^l^ Describes Old World monkeys (OWM) that engage in >68% terrestrial quadrupedal locomotion (Rose, 1977; Hunt, 1991; Fleagle, 1999).

**Table 2:** Fossil hominoid sample used in this study

**Carpal**	**Taxon**	**Specimen**
**Scaphoid**	*Proconsul africanus*	KNM SO 999*
	*Proconsul heseloni*	KNM RU 2036*, C14*
	*Oreopithecus bambolii*	Basel 26*
	*Australopithecus* sp.	StW 618
	*Australopithecus sediba*	MH2 UW 88-158
	*Homo habilis*	OH7
	*Homo neanderthalensis*	Kebara 2
**Lunate**	*Proconsul nyanzae*	KNM RU 15100*
	*Proconsul heseloni*	KNM RU 2036*, C22*
	*Australopithecus sediba*	MH2 UW 88-159
	*Homo neanderthalensis*	Kebara 2, Amud 1
**Triquetrum**	*Proconsul nyanzae*	KNM RU 15100*
	*Australopithecus sediba*	MH2 UW 88-157
	*Au. robustus* or early *Homo*	SKX 3498
	*Homo neanderthalensis*	Kebara 2, Amud 1
**Capitate**	*Proconsul africanus*	KNM CA 409*
	*Proconsul heseloni*	KNM RU 2036*, KNM RU 1907*, C25*, C26*, C28*
	*Afropithecus turkanensis*	KNM 18365*
	*Sivapithecus indicus*	GSP Y500 17119*
	*Rudapithecus hungaricus*	RUD 167
	cf. *Australopithecus*	KNM-WT 22944-H**
	*Australopithecus afarensis*	AL 333-40
	*Australopithecus africanus*	TM 1526
	*Australopithescus sediba*	MH2 UW 88-105
	*Homo neanderthalensis*	Kebara 2, Amud 1
**Hamate**	*Proconsul heseloni*	KNM RU 2036*
	*Sivapithecus paravada*	NG Y311 940
	*Oreopithecus bambolii*	Basel 36
	cf. *Australopithecus*	KNM-WT 22944-I**
	*Australopithecus afarensis*	AL 333-50
	*Australopithecus sediba*	MH2 UW 88-106
	*Homo neanderthalensis*	Kebara 2

*Measurements taken from cast.

**Metric data derived from [65,87].

## Results and discussion

We investigated the evolution of wrist bone morphology across a sample of extant and fossil haplorrhines using a phylogenetically-integrated approach that quantifies evolutionary changes along individual branches of an independently-derived phylogeny. Although the eight or nine carpal bones that comprise the primate wrist can be described as functioning as an anatomical module linking the forearm to the hand, our results demonstrate that the morphology of individual wrist bones follows different evolutionary pathways, supporting the view that the wrist is better considered as an integrated, complex system of joints in which individual bones have a degree of functional and evolutionary independence [46-52]. The evolutionary morphological changes in some wrist bones are consistent with similarities in locomotor behaviour shared across taxa (scaphoid, triquetrum and capitate) while others (lunate and hamate) indicate taxon- or clade-specific synapomorphic patterns. Generally, within our sample, hominoids display more morphological variation, particularly in the hamate (PC1: *F* = 7.25, *P* < 0.001; PC2: *F* = 7.92, *P* < 0.001), triquetrum (PC1: *F* = 5.02, *P* < 0.001; PC2: *F* = 2.85, *P* = 0.017), lunate (PC1: *F* = 4.34, *P* < 0.005; PC2: *F* = 1.23, *P* = 0.67), and capitate (PC1: *F* = 1.79, *P* = 0.16; PC2: *F* = 11.64, *P* < 0.001) than New World monkeys (NWM) and Old World monkeys (OWM), supporting previous analyses of the wrist [53-56]. The increased variation in the evolutionary morphology of hominoids suggests that this clade has been subject to increased selective pressures on wrist morphology compared with other primates. Altogether, we find evidence for several instances of independent evolution in wrist bone morphology across haplorrhines, and particularly within hominoids.

**Figure 2:**
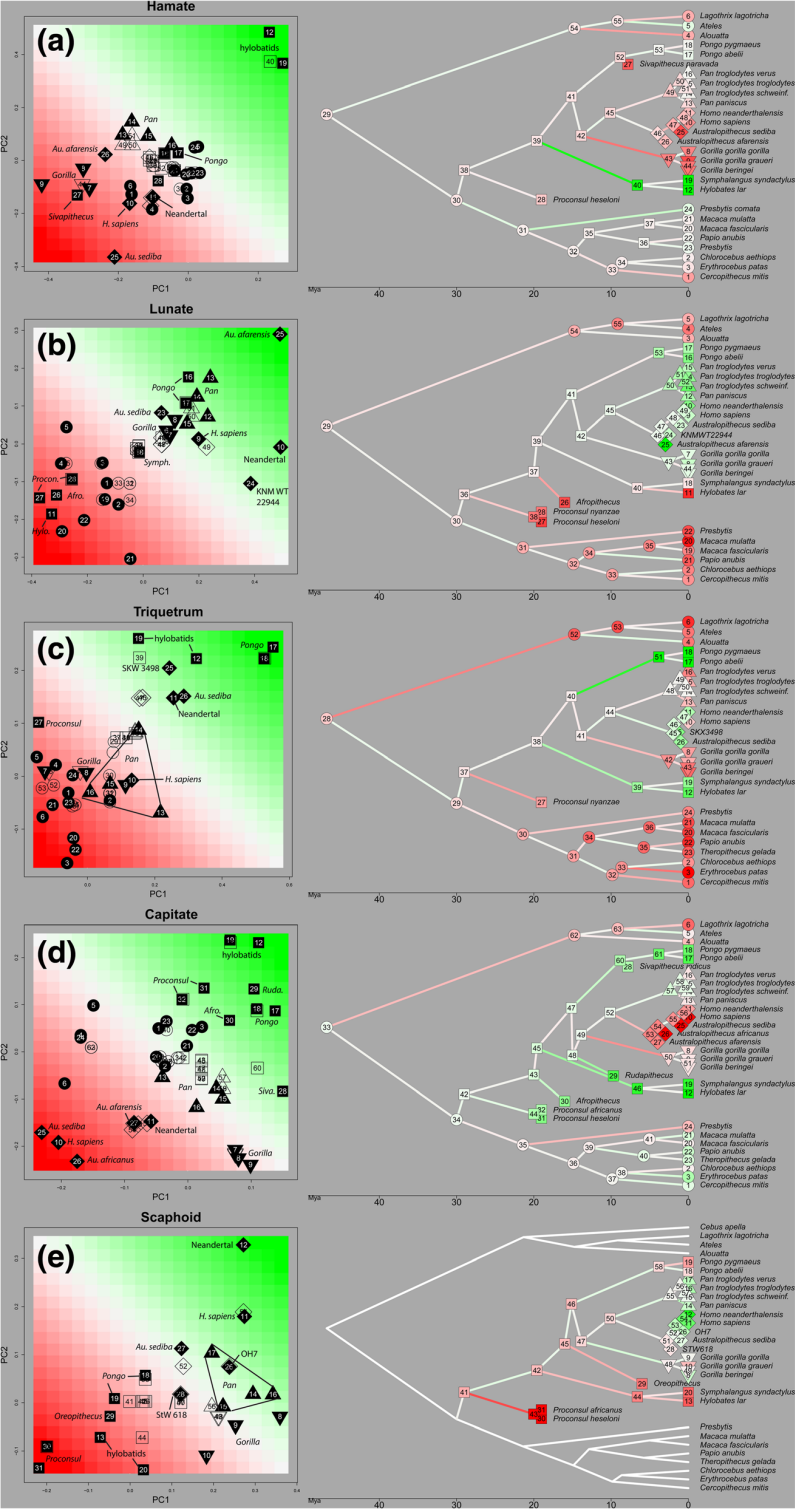
**Results of the phylogenetic principal components analysis (PC1 and PC2) of wrist variables, and the estimated ancestral states (nodes) and rates (branches) plotted in morphospace (left) and on a phylogenetic tree (right) for the (a) hamate, (b) lunate, (c) triquetrum, (d), capitate and (e) scaphoid.** Full symbols in the plot represent (observed) tip values in the tree; empty symbols in the plot represent internal nodes (estimated ancestral values) in the tree. In both the plot and tree, circles indicate New World monkeys; squares, hominoid ancestors and extant Asian apes; triangles, *Pan*; reverse triangles, *Gorilla*; diamonds, hominins. Colour gradients in the plot are allocated according to the variation of PC scores. Branch colour and hue in the tree indicate direction of change between ancestor and descendant. Figure 2e presenting the scaphoid results shows hominoids only (see Additional file 3 for an additional plot and tree including all haplorrhines).

The evolutionary morphology of the hamate (Figure 2a, Table 3) sets apart the hylobatids, while the lunate (Figure 2b, Table 3) distinguishes the great apes from other primates. These bones thus seem to reflect instances of autapomorphic morphological evolution. This is particularly true in the hamate, in which only hylobatids display a derived, extremely proximodistally long but dorsopalmarly short hamate. This result is consistent with previous studies [46,50,51,57] and the elongation of other skeletal elements of the hylobatid forelimb, which is considered advantageous for brachiation [58]. Elongation of the hamate is not found, however, in semi-brachiator *Ateles*, largely because *Ateles* lacks a distally extended hamulus that is typical of hylobatids [55,57]. This lack of convergence may reflect the more varied locomotor repertoire of *Ateles*, such that they do not engage in the ricochetal brachiation of hylobatids, make use of a prehensile tail during suspensory locomotion, and also engage in a substantial amount (21% of locomotor time) of quadrupedal walking and running [59]. Previous studies have noted that most of the morphological convergence in the forelimb between *Ateles* and hylobatids can be found in the shoulder and elbow [37,60,61] and that overall brachiating features are not as pronounced in *Ateles*[54,60,62].

**Table 3:** Descriptions of the wrist variables, their respective loadings on PC1 and PC2, and the variance explained by each PC

**Carpal**	**Variable**	**Description**	**PC1**	**PC2**
**Hamate**	LHB-H	Maximum proximodistal length of hamate body (excluding hamulus)	**0.846**	0.032
	HHTF	Maximum dorsopalmar height of hamate triquetrum facet	0.321	**−0.399**
	LHB	Maximum proximodistal length of hamate body	0.082	**0.970**
	LHTF	Maximum proximodistal length of hamate triquetrum facet	0.02	0.094
	HHB	Maximum dorsopalmar height of hamate body (including hamulus)	−0.273	−0.034
	HHB-H	Maximum dorsopalmar height of hamate body (excluding hamulus)	**−0.660**	−0.234
	BHB	Maximum mediolateral breadth of hamate body	**−0.935**	−0.015
Variance explained			55.23%	22.39%
**Lunate**	BLRF	Maximum mediolateral breadth of lunate radial facet	**0.884**	−0.114
	HLRF	Maximum dorsopalmar height of lunate radial facet	**0.747**	0.024
	BLCF	Maximum mediolateral breadth of kunate capitate facet	0.452	−0.361
	BLB	Maximum mediolateral breadth of lunate body	0.318	**0.741**
	HLTF	Maximum dorsopalmar height of lunate triquetrum facet	0.235	−0.09
	HLCF	Maximum dorsopalmar height of lunate capitate facet	0.188	0.388
	LLTF	Maximum proximodistal length of lunate triquetrum facet	−0.061	−0.253
	HLB	Maximum dorsopalmar height of lunate body	−0.118	**0.962**
	LLB	Maximum proximodistal length of lunate body	−0.393	0.304
	HLSF	Maximum dorsopalmar height of lunate scaphoid facet	**−0.673**	**−0.571**
	LLSF	Maximum proximodistal length of lunate scaphoid facet	**−0.952**	−0.091
Variance explained			53.99%	22.61%
**Triquetrum**	HTHF	Maximum dorsopalmar height of triquetrum hamate facet	**0.907**	0.219
	BTHF	Maximum mediolateral breadth of triquetrum hamate facet	0.529	0.019
	BTB	Maximum mediolateral breadth of triquetrum body	0.385	0.14
	HTLF	Maximum dorsopalmar height of triquetrum lunate facet	0.033	0.2
	LTB	Maximum proximodistal length of triquetrum body	−0.036	−0.061
	LTLF	Maximum proximodistal length of triquetrum lunate facet	−0.08	**−0.972**
	HTB	Maximum dorsopalmar height of triquetrum body	**−0.697**	0.258
Variance explained			72.75%	12.96%
**Capitate**	BCN	Minimum mediolateral breadth of capitate neck	**0.987**	−0.121
	BCB	Maximum mediolateral breadth of capitate body	0.165	0.367
	BCPF	Maximum mediolateral breadth of capitate proximal facet	0.035	**−0.834**
	LCB	Maximum proximodistal length of capitate body	−0.096	**0.972**
	HCPF	Maximum dorsopalmar height of capitate proximal facet	**−0.859**	−0.107
	HCB	Maximum dorsopalmar height of capitate body	**−0.971**	0.087
Variance explained			70.93%	22.14%
Scaphoid*	LSB	Maximum proximodistal length of scaphoid body	**0.951**	−0.089
	HSB	Maximum dorsopalmar height of scaphoid body	0.352	−0.075
	LSRF	Maximum proximodistal length of scaphoid radial facet	0.243	0.146
	HSRF	Maximum dorsopalmar height of the scaphoid radial facet	0.186	−0.13
	BSB	Maximum mediolateral breadth of scaphoid body	−0.045	**0.936**
	HSLF	Maximum dorsopalmar height of scaphoid lunate facet	−0.056	**−0.36**
	LSLF	Maximum proximodistal length of scaphoid lunate facet	**−0.693**	−0.129
Variance explained			65.12%	16.32%

Variables with the highest positive and negative loadings are highlighted in bold. *Note that the os centrale is fused to the scaphoid in *Pan*, *Gorilla*, humans and fossil hominins and thus measurements such as the maximum height of the scaphoid body (HSB) and height of the scaphoid’s lunate facet (HSLF) are not necessarily developmentally or morphologically homologous across our primate sample (i.e., the fused os centrale is included in the measurement for these taxa, but not for the other primates in the sample).

Conversely, *Gorilla, Sivapithecus* and, particularly, *Au. sediba* are derived from all of the remaining taxa, evolving in parallel a proximodistally shorter and mediolaterally broader hamate. *Au. sediba* is further distinguished with an even proximodistally shorter hamate body but comparatively dorsopalmarly tall triquetral facet. This pattern is consistent with increased compressive loading during quadrupedal [34,63,64] and potentially knuckle-walking [33] locomotor behaviours shared between *Gorilla* and *Sivapithecus*. *Pan* does not demonstrate the same morphology as *Gorilla*, perhaps due to the engagement in more arboreal behaviours (Table 1), but this variation in hamate morphology between *Pan* and *Gorilla* has been noted previously [18,50,65]. Although previous comparative research on forelimb morphology has concluded that *Pan* is unique compared with other hominoids and derived from the common *Pan*-human ancestor [31,66-68], our phylogenetic analysis suggests, when it comes to hamate morphology, that *Pan* retains a plesiomorphic condition and that *Gorilla* is derived. Reasons as to why *Au. sediba* demonstrates such a derived pattern compared with other fossil and extant hominines requires further investigation.

In the lunate (Figure 2b, Table 3), NWMs, OWMs, *Hylobates* and Miocene apes, have evolved from the ancestral haplorrhine or hominoid condition towards a proximodistally shorter and mediolaterally narrower lunate with a smaller (both proximodistally and mediolaterally) radial facet and a larger (both proximodistally and dorsopalmarly) scaphoid facet. *Gorilla* generally retains the ancestral hominid condition, while *Pan* and *Pongo* have evolved in parallel a slightly dorsopalmarly taller and mediolaterally broader lunate with a larger (both proximodistally and mediolaterally) radial facet but smaller (both proximodistally and dorsopalmarly) scaphoid facet. This shared morphology in *Pan* with *Pongo* may reflect larger loading of the radiolunate joint in adducted position during climbing [17,69]. Although this pattern falls in contrast to previous qualitative and quantitative descriptions of lunate morphology in which the *Pongo* lunate is noted as being particularly broad and distinguished from that of knuckle-walking African apes [47,54,70], relative to size, *Pongo* and African apes do not significantly differ in the breadth of the lunate [71].

Interestingly, all fossil hominins, apart from *Au. sediba*, which displays a plesiomorphic hominoid morphology, display derived lunate morphology; *Au. afarensis*, cf., *Australopithecus* KNM-WT 22944-J and *H. neanderthalensis* all have a much larger radial facet and smaller scaphoid facet, compared with extant hominoids (including *H. sapiens*). In *Australopithecus*, this may reflect continued use of climbing [17,69], although the reasons for its parallel development in *H. neanderthalensis* or its absence in *Au. sediba* require further investigation.

Triquetrum and capitate (Figure 2c,d, Table 3) evolutionary morphology indicates several occurrences of parallel evolution. In both wrist bones, suspensory/brachiating *Pongo* and hylobatids display parallel evolution from their respective ancestral conditions towards a morphology that is not identical but consistent with greater mobility at the midcarpal joints [72,73]. In the triquetrum, hylobatids and *Pongo* both demonstrate a short proximodistal length of the lunate facet, while *Pongo* is further distinguished by dorsopalmarly shorter triquetrum facet but taller hamate facet. In contrast, hominines display various patterns of parallel evolution from the ancestral condition in the opposite direction (dorsopalmarly taller triquetrum but shorter hamate facet, and proximodistally longer lunate facet). These patterns are consistent with the uniquely narrow (and distally-positioned) *Pongo* triquetrum, compared with the pyramidal morphology and spiral triquetrum-hamate articulation typical of African apes, which are considered related to greater compressive loading and stability [47,50,57,70].

Interestingly, in the triquetrum, the ancestral hominin condition is most similar to the “suspensory/brachiating” morphology, such that all fossil hominins, including *H. neanderthalensis*, are more similar to Asian apes than to *H. sapiens* or African apes [74]. This suggests that the African ape-like triquetrum morphology of *H. sapiens* may have evolved only recently in parallel with *Pan* and *Gorilla.* The pyramidal triquetrum of *H. sapiens* could be related to increased loading on the ulnar side of the wrist and fifth finger during habitual tool-making and tool-use [75], although the loads experienced by the wrist bones during these manipulative behaviours remain to be experimentally tested. This hypothesis is not supported by the habitual, complex tool-use in *H. neanderthalensis*, which retains a primitive triquetrum morphology, although subtle differences in the power and precision grips between *H. neanderthalensis* and *H. sapiens* have been proposed [26,76].

In the capitate (Figure 2d, Table 3), *Pan* retains the ancestral hominoid condition, while *H. sapiens* and fossil hominins have evolved in parallel with *Gorilla* a proximodistally shorter length of the capitate body with a mediolaterally broader proximal facet. Therefore, unlike the triquetrum, the similarities in capitate morphology shared between *H. sapiens* and *Gorilla* evolved earlier, appearing at least by the time of *Au. afarensis* (4 Ma) and being retained in all later hominins. *Sivapithecus* has also evolved in parallel a capitate morphology that is broadly similar to African apes, as has been previously described [33]. In contrast, extant *Pongo* and, particularly, hylobatids, demonstrate an extreme version of the ancestral hominoid condition seen in *Proconsul* and *Afropithecus*, with a proximodistally longer but dorsopalmarly shorter capitate body and smaller proximal facet (both mediolaterally and dorsopalmarly). Suspensory *Rudapithecus* also converges on the *Pongo* condition, which is consistent with previous functional interpretations of its postcranial skeleton [77,78].

Scaphoid evolutionary morphology (Figure 2e, Table 3, Additional file 2) is more complex compared with the other wrist bones. This complexity can be at least partly explained by variation in locomotor behaviours and, perhaps also related to function, by the fusion of the os centrale to the scaphoid in hominines [16,17,29]. Asian apes, as well as *Oreopithecus,* retain the ancestral hominoid condition of a smaller scaphoid body (both proximodistally and mediolaterally), reflecting the absence of scaphoid-centrale fusion, and a larger (both proximodistally and dorsopalmarly) lunate facet, possibly associated with greater mobility among the radial wrist bones [79]. However, *Proconsul* demonstrates the extreme of this pattern, yet is reconstructed as an arboreal quadruped [57,58]. Among hominines, there is parallel evolution each in *Gorilla*, *Pan*, and fossil hominins (*H. sapiens*, *H. neanderthalensis,* OH7 and *Au. sediba*) towards a smaller lunate articulation and a larger scaphoid body. Although the latter reflects fusion of the os centrale to the scaphoid body, this does not imply that scaphoid-centrale fusion occurred in parallel since other fossil hominins also have a fused os centrale and yet comparatively smaller scaphoid bodies. *Australopithecus* sp. StW 618 retains a morphology that is more similar to the ancestral hominoid condition than the more *Pan*-like morphology of *H. habilis* and (to a lesser extent) *Au. sediba*. The *H. sapiens* scaphoid morphology shares morphological similarities with both *Au. sediba* and *H. habilis* and thus it remains unclear which may better represent the ancestral condition for later *Homo*. Altogether, this analysis demonstrates substantial parallel evolution in scaphoid morphology across hominines.

## Conclusions

Analysis of hominoid wrist morphology within a broad, phylogenetic context confirms numerous instances of parallel evolution across haplorrhines and among extant hominoids that have been recognised by previous studies [8,50,54], including shared morphology between *Pongo* and hylobatids [8,57], *Pongo* and *Rudapithecus*[77,78], and *Sivapithecus* and African apes [33,63,64]. However, this analysis also reveals substantial parallel evolution within extant and fossil hominines, some of which has been previously discussed [18,20,30] but not demonstrated within a robust phylogenetic analysis. There is no consistent pattern of evolutionary change within taxa across all five wrist bones. Instead, results indicate that extant and fossil variation in wrist morphology is best explained by a mixture of parallel and synapomorphic changes across different wrist bones, each potentially influenced by both function (i.e., corresponding to similar locomotor behaviours) and phylogeny.

The occurrence of different processes of evolution in combination with a higher variation in wrist bone morphology in hominoids [53-56,80] indicates an increased selective pressure and a higher evolvability of hominoid wrist bone morphology in comparison with other primates. This result is to some degree not surprising given the large range of variation in body size and locomotor behaviours that typify the hominoid clade (Table 1). In addition, within the context of hominoid fossil record, extreme suspensory behaviour has likely evolved multiple times in hylobatids, *Pongo*, *Oreopithecus* and potentially *Rudapithecus*[8,61] allowing for both the development of similar and different morphological features to meet similar same functional demands. In contrast, individual hominoid taxa all retain some aspect of primitive ancestral morphology in some wrist bones, while being derived in others. Recent work [31,67,68] has suggested that extant African apes, and particularly *Pan*, are too derived to provide informative morphological comparisons when interpreting hominin morphology. Our results suggest that while this may be true for the scaphoid and triquetrum morphology, African apes retain ancestral morphologies in the other wrist bones and thus should not be discounted within an evolutionary context. These results highlight the importance of incorporating a broad comparative sample of species, phylogenetic information and time within morphological studies and put into context the use of particular extant species for morphological comparisons when interpreting hominin morphology.

Much debate remains over the locomotor behaviour of the pre-bipedal common ancestor of *Homo* and *Pan*[8,17,18,20,29,81], in which the shared morphology of the wrist has played a central role [16-19]. One model envisions the pre-bipedal ancestor as a knuckle-walker that had already come down to the ground, similar to the locomotor behavior used by African apes [17,29,81,82]. In the alternative model, early human bipedalism is seen as having evolved from a more generalized arboreal, climbing-oriented ancestor, a mode of locomotion that is used by all living apes [18,23,83-85]. Each scenario has important and profoundly different implications for understanding the evolution of ape and human locomotion. The phylogenetically-integrated approach applied here offers new insight into this debate; We reveal a substantial amount of parallel evolution in wrist morphology among extant *Pan, Gorilla* and *H. sapiens*, as well as fossil hominins, including in the morphologies of the scaphoid, triquetrum and capitate that are typically considered advantageous for accommodating increased compressive loading during knuckle-walking [17,47,50]. These results may add further support to hypotheses that both knuckle-walking behavior [18,20] and the African ape-like features of hominins [27] have evolved in parallel within hominines. However, this signal is not consistent across all wrist bones included in this study and the functional association between these features and knuckle-walking locomotion requires further testing, especially given the variation in wrist and hand morphology across African apes [18,86]. Further phylogenetically-integrated analyses of other skeletal elements within the forelimb are also needed to robustly test these hypotheses.

In conclusion, we demonstrate how a combination of parallel and synapomorphic evolution across different wrist bones best explains the morphological diversity observed in extant and fossil hominoids. These results exemplify how the evolution of a single skeletal anatomical unit - the wrist - can be shaped by a diverse pattern of evolutionary trends, with each bone having a degree of functional and/or evolutionary independence. The diversity in evolutionary patterns across the wrist bones revealed here contributes to explaining the mosaic nature of the wrist and other postcranial morphology observed in recent fossil finds [3,4,87,88].

## Methods

### Sample

Morphometric data were collected from adult individuals of a diverse sample of haplorrhines of varying body size and locomotor behaviours (Table 1). All individuals were free of obvious pathologies and considered adult based on dental eruption and/or epiphyseal fusion throughout the associated skeleton. Fossil specimens include Miocene hominoids and Plio-Pleistocene hominins (Table 2). Miocene hominoid genera include early Miocene *Proconsul* (20–17 Ma) and *Afropithecus* (17.5-17 Ma), mid- to late Miocene *Sivapithecus* (12.5-8.5 Ma), and late Miocene *Rudapithecus* (10 Ma) and *Oreopithecus* (9–7 Ma) [8,89]. The postcranial skeletons of *Proconsul heseloni* and *P. nyanzae* are well-represented and the locomotor behaviour of both has been reconstructed generally as pronograde, above-branch, arboreal quadrupedalism using a grasping, palmigrade hand posture [8,90,91]. Morphological similarities between the few postcranial remains of *Afropithecus* and *P. nyanzae*, suggest that the former was also an arboreal quadruped [92]. *Sivapithecus* postcranial remains reveal a suite of features that are unlike any extant primate analogue [34,63,64,93,94]. Generally it is considered to have been mainly arboreal, emphasizing pronograde, arboreal quadrupedalism [63,64,93,95], but more recent research has proposed that vertical climbing [34] and perhaps knuckle-walking [33] were part of the *Sivapithecus* locomotor repertoire. *Rudapithecus* postcranial remains demonstrate distinct similarities to extant great apes [77], including strong phalangeal curvature [78], all of which emphasize suspensory locomotion. The *Oreopithecus* postcranium is unique, with several morphological features suggesting highly specialized orthograde behaviors, including suspension [96,97] and perhaps bipedalism [98], while some have suggested its hand was capable of hominin-like grasping dexterity [99], but see [100].

Table 3 lists the measurements for each bone, which are depicted graphically in the supplementary material (Additional file 3). Mean and standard deviation for each variable, wrist bone and each taxon are given in Additional file 1. These metrics were chosen to quantify the overall size of each wrist bone and their articular facets in a manner that could be reliably measured in a diverse sample of morphologies across different primates. All measurements were taken by one observer (T.L.K.) and on the right side, unless unavailable (then measurements were taken on the left). Intraobserver measurement error was tested on n = 25 specimens on three separate occasions. Measurement errors were calculated using methods outlined by White [101], and the average error was less than 1% for most variables, although closer to 1.5% for measurement of the length of the scaphoid body (LSB), length and breadth of lunate’s radial facet. This measurement error is consistent with other morphometric studies of the hand [102]. Measurements for all Miocene hominoid taxa were taken on casts, except for *Sivapithecus parvada* (NG Y311 940) and *Rudapithecus* (RUD 167). Measurements for all Plio-Pleistocene hominin specimens were taken on the original fossils, except those of cf. *Australopithecus* KNM WT 22944, which were taken from casts (Table 2).

Since body weight information is most often not available with museum specimens, all of the raw measurements for each carpal bone were used to create a geometric mean. The geometric mean was used a size-correcting variable [103-105].

### Phylogeny

The primate molecular phylogeny was taken from the 10 k Trees Project (version 3) [106]. Divergence dates for great apes were amended following [107]. Fossil species were placed onto the phylogeny following the best solution given the divergence dates of the molecular phylogeny [106] and the inferred taxonomic divergence and time of last occurrence of the fossils following recently published information [3,78,108].

### Statistical procedure

Size-corrected morphological measurements were used as input to a phylogenetically weighted principal components analysis in order to minimize type I error in statistical estimators [109,110]. Output scores represent species values that are weighted for phylogenetic distance. The variation of PC scores (for both observed and inferred ancestral species) between hominoids and other haplorrhines for each PC for each bone were compared using an F-test for equality of variance.

### Inferring variable rates and ancestral estimates

The output scores of the phylogenetically-weighted principal components analyses were used to estimate rates of change and ancestral values. Phylogenetic comparative methods infer evolutionary trends based on variation of a trait across different species and a phylogenetic tree that describes the relatedness between those species [111]. To infer changes on ancestral branches, models of evolution are used that lay out the principles to ‘count back in time’ [11,112]. We use a method (‘Independent Evolution’ , ‘IE’) that is based on the principles of an Adaptive Peak model of evolution [112] and allows ancestral state and variable rate estimation for individual branches of a phylogenetic tree [12,45]. This method incorporates aspects of more traditionally used models (Brownian Motion and Ornstein-Uhlenbeck) as special cases. The assumptions of the IE method consider that extant variation is the necessary result of foregone changes, and thus that extant trait variation bears crucial information on evolutionary history. IE infers evolution based only on observed (extant and/or fossil) variation and phylogenetic relatedness (no a priori model parameters), and therefore has less stringent data assumptions and a decreased reliance on a prior model assumptions [113].

### Phylogenetic mapping

To visualize the temporal origin and rate of underlying morphological changes for all individual branches of the phylogenetic tree, we mapped the inferred rates and ancestral values back onto the phylogeny in a colour-coded manner. Plots in Figure 2 indicate the PCA-scores for all observed (full symbols) and estimated ancestral values (empty symbols). Colour codes (shades of green and red) were chosen to indicate a gradual differentiation between PC1 and PC2. Corresponding colour codes were used to represent the observed and estimated values in the tree. The colour hue for the nodal values hereby represents their location in PC1-PC2 morphospace. Colour codes for the branches in the tree relate to the rate of change in either direction of the morphospace (green or red-coded in the plot). The colour hue for the branches in the tree corresponds to the absolute value of the rate of change (with a more intense colour for higher rates of change).

## Competing interests

The authors declare that they have no competing interests.

## Authors’ contributions

All authors participated in conceiving and designing the study. TLK provided data. JBS performed the statistical analyses. All authors wrote the paper. All authors read and approved the final manuscript.

## Supplementary Material

Additional file 1 **Mean (above) and standard deviation (below) for each carpal variable and the geometric mean (geomean) in each taxon for the (a) hamate, (b) lunate, (c) triquetrum, (d) capitate and (e) scaphoid.** Variable acronyms the same as in Table 1. Click here for file

Additional file 2 **Measurements for each wrist bone used in this analysis, shown on human bones as an example.** Acronyms are the same as described in Table 3. Click here for file

Additional file 3 **Results of the phylogenetic principal component analysis (PC1 and PC2) on the scaphoid wrist variables, including all haplorrhine taxa, and the estimated ancestral states (nodes) and rates (branches) plotted in morphospace (left) and on a phylogenetic tree (right).** Symbols and colour gradient the same as described in Figure 2. Click here for file
