# Supplementary material for: Different evolutionary pathways underlie the morphology of wrist bones in hominoids
Source: BMC Evol Biol. 2013 Oct 23;13:229. doi: 10.1186/1471-2148-13-229 (PMC4015765; doi:10.1186/1471-2148-13-229)
Supplement: Additional file 1 — Mean (above) and standard deviation (below) for each carpal variable and the geometric mean (geomean) in each taxon for the (a) hamate, (b) lunate, (c) triquetrum, (d) capitate and (e) scaphoid. Variable acronyms the same as in Table 1. [file 1471-2148-13-229-S1.docx]

**ESM 1.** Mean (above) and standard deviation (below) for each carpal variable and the geometric mean (geomean) in each taxon for the (a) hamate, (b) lunate, (c) triquetrum, (d) capitate and (e) scaphoid. Variable acronyms the same as in Table 1.

**(a) Hamate**

| **Taxon** | **LHB** | **LHB-H** | **HHB** | **HHB-H** | **BHB** | **HHTF** | **LHTF** | **GEOMEAN** |
| --- | --- | --- | --- | --- | --- | --- | --- | --- |
| *Alouatta* | 9.34 | 8.87 | 7.21 | 5.90 | 7.93 | 5.77 | 9.24 | 7.60 |
|  | 0.76 | 0.90 | 0.88 | 0.62 | 0.67 | 0.53 | 0.59 | 0.51 |
| *Ateles* | 12.67 | 11.61 | 7.51 | 6.54 | 9.12 | 5.92 | 11.31 | 8.87 |
|  | 2.04 | 1.80 | 0.96 | 0.43 | 0.89 | 0.73 | 1.97 | 0.97 |
| *Au. afarensis* | 20.95 | 18.23 | 18.59 | 12.22 | 15.11 | 9.84 | 13.36 | 15.02 |
| *cf. Australopithecus* | 22.58 | 16.55 | 19.56 | 12.29 | 14.72 | 9.07 | 14.46 | 15.03 |
| *Au. sediba* | 16.82 | 15.77 | 18.29 | 11.93 | 14.00 | 13.09 | 14.48 | 14.75 |
|  | 1.30 | 0.18 | 1.12 | 0.23 | 0.16 | 1.95 | 1.09 | 0.19 |
| *Cercopithecus mitis* | 9.46 | 9.30 | 8.27 | 6.22 | 8.18 | 4.86 | 6.71 | 7.37 |
|  | 1.05 | 1.04 | 0.63 | 0.50 | 0.61 | 0.73 | 0.43 | 0.49 |
| *Chlorocebus aethiops* | 8.57 | 8.43 | 6.81 | 5.44 | 5.93 | 4.73 | 7.09 | 6.57 |
|  | 0.98 | 0.80 | 1.01 | 0.83 | 1.18 | 0.72 | 1.55 | 0.98 |
| *Erythrocebus patas* | 9.78 | 10.15 | 8.08 | 6.19 | 7.47 | 5.70 | 8.03 | 7.75 |
|  | 1.21 | 1.50 | 1.59 | 1.08 | 1.44 | 1.31 | 1.46 | 1.33 |
| *Gorilla beringei* | 26.65 | 21.00 | 25.66 | 16.49 | 18.67 | 13.86 | 19.15 | 19.71 |
|  | 3.41 | 2.43 | 4.19 | 2.30 | 2.73 | 1.34 | 2.93 | 2.33 |
| *G. gorilla gorilla* | 32.05 | 22.69 | 29.01 | 17.21 | 21.46 | 16.06 | 21.45 | 22.17 |
|  | 4.14 | 3.57 | 4.63 | 2.95 | 2.94 | 2.26 | 3.34 | 2.93 |
| *G. gorilla graueri* | 25.95 | 18.42 | 26.08 | 16.64 | 18.89 | 12.75 | 18.86 | 19.11 |
|  | 1.79 | 2.11 | 4.44 | 2.30 | 1.52 | 1.17 | 1.11 | 1.67 |
| *Homo sapiens* | 21.45 | 20.44 | 22.38 | 13.56 | 15.59 | 12.31 | 17.32 | 17.15 |
|  | 2.37 | 2.03 | 2.79 | 1.47 | 1.80 | 1.59 | 2.11 | 1.73 |
| *Homo neanderthalensis* | 24.45 | 22.52 | 24.62 | 13.30 | 16.14 | 14.70 | 19.32 | 18.79 |
| *Hylobates lar* | 17.03 | 13.68 | 9.80 | 6.01 | 6.70 | 5.40 | 10.43 | 9.09 |
|  | 1.19 | 0.99 | 0.89 | 0.55 | 0.51 | 0.41 | 1.08 | 0.48 |
| *Lagothrix lagotricha* | 8.86 | 8.86 | 7.77 | 5.67 | 7.77 | 4.41 | 9.08 | 7.26 |
|  | 0.67 | 0.67 | 0.89 | 0.41 | 0.76 | 0.47 | 0.70 | 0.49 |
| *Macaca fascicularis* | 8.71 | 8.57 | 6.72 | 5.15 | 6.20 | 4.44 | 7.08 | 6.51 |
|  | 0.51 | 0.37 | 0.42 | 0.77 | 0.47 | 0.52 | 0.40 | 0.38 |
| *Macaca mulatta* | 11.53 | 11.42 | 9.21 | 6.81 | 8.60 | 5.62 | 9.34 | 8.67 |
|  | 0.91 | 0.84 | 0.93 | 0.77 | 0.93 | 0.92 | 1.09 | 0.83 |
| *Pan paniscus* | 25.23 | 18.12 | 18.35 | 12.82 | 15.71 | 10.88 | 16.23 | 16.21 |
|  | 2.04 | 1.25 | 1.18 | 0.66 | 1.27 | 1.66 | 1.55 | 1.01 |
| *P. troglodytes schweinfurthii* | 27.97 | 19.93 | 19.23 | 13.81 | 16.82 | 11.35 | 18.51 | 17.57 |
|  | 2.19 | 1.36 | 2.10 | 0.64 | 1.56 | 1.05 | 1.67 | 1.04 |
| *P. troglodytes troglodytes* | 28.03 | 21.69 | 20.85 | 14.25 | 16.46 | 12.25 | 19.27 | 18.31 |
|  | 3.21 | 2.90 | 2.09 | 1.49 | 2.02 | 1.48 | 1.95 | 1.69 |
| *P. troglodytes verus* | 28.76 | 22.53 | 21.03 | 14.56 | 16.04 | 13.73 | 18.20 | 18.60 |
|  | 3.36 | 1.75 | 2.26 | 1.74 | 1.76 | 2.43 | 3.60 | 1.76 |
| *Papio anubis* | 16.66 | 16.40 | 12.70 | 9.68 | 11.63 | 8.53 | 13.92 | 12.42 |
|  | 2.03 | 1.97 | 1.92 | 1.04 | 1.47 | 1.32 | 1.76 | 1.39 |
| *Pongo abelii* | 24.31 | 22.30 | 19.11 | 14.21 | 14.74 | 10.89 | 18.42 | 17.11 |
|  | 3.28 | 2.32 | 2.68 | 1.75 | 1.75 | 1.27 | 2.47 | 1.82 |
| *Pongo pygmaeus* | 25.43 | 22.31 | 20.92 | 14.13 | 15.08 | 11.68 | 19.44 | 17.80 |
|  | 4.20 | 3.16 | 3.35 | 1.83 | 1.88 | 1.71 | 2.84 | 2.32 |
| *Presbytis* | 10.59 | 10.27 | 7.65 | 6.01 | 7.11 | 4.58 | 8.05 | 7.48 |
|  | 0.61 | 0.09 | 0.40 | 0.18 | 0.47 | 0.25 | 0.58 | 0.24 |
| *Proconsul heseloni* | 12.82 | 12.66 | 10.46 | 7.42 | 10.57 | 6.54 | 10.81 | 9.91 |
| *Sivapithecus paravada* | 26.58 | 22.16 | 23.34 | 20.42 | 20.90 | 12.78 | 17.31 | 20.04 |
| *Symphalangus syndactylus* | 18.78 | 15.90 | 10.15 | 7.12 | 8.11 | 6.99 | 12.68 | 10.62 |
|  | 1.65 | 1.33 | 0.77 | 0.80 | 0.91 | 0.77 | 2.46 | 0.99 |
| *Theropithecus gelada* | 14.08 | 14.08 | 10.81 | 8.57 | 9.43 | 7.08 | 10.18 | 10.31 |
|  | 0.92 | 0.92 | 0.86 | 0.49 | 0.66 | 0.92 | 0.56 | 0.60 |

**(b) Lunate**

| **Taxon** | **LLB** | **HLB** | **BLB** | **HLSF** | **LLSF** | **HLCF** | **BLCF** | **HLRF** | **BLRF** | **HLTF** | **LLTF** | **GEOMEAN** |
| --- | --- | --- | --- | --- | --- | --- | --- | --- | --- | --- | --- | --- |
| *Alouatta* | 7.71 | 6.59 | 4.56 | 5.95 | 3.61 | 5.93 | 3.45 | 5.60 | 5.38 | 3.44 | 4.05 | 4.93 |
|  | 0.41 | 0.31 | 0.29 | 0.50 | 0.42 | 0.31 | 0.31 | 0.49 | 0.41 | 0.24 | 0.52 | 0.21 |
| *Ateles* | 9.86 | 8.53 | 5.74 | 7.38 | 5.46 | 6.88 | 4.14 | 7.24 | 5.95 | 4.11 | 4.45 | 6.08 |
|  | 1.03 | 0.52 | 0.77 | 0.73 | 0.59 | 0.55 | 0.62 | 0.66 | 1.08 | 0.50 | 0.69 | 0.38 |
| *Au. sediba* | 12.36 | 12.81 | 9.93 | 9.28 | 6.45 | 9.10 | 5.04 | 11.90 | 11.43 | 6.74 | 7.84 | 8.99 |
| *cf. Australopithecus* | 11.60 | 15.80 | 14.10 | 15.00 | 6.00 | 12.40 | 9.80 | 15.40 | 14.20 | 8.40 | 8.30 | 11.42 |
| *Au. afarensis* | 14.00 | 16.50 | 16.20 | 11.50 | 3.50 | 11.70 | 8.50 | 15.00 | 15.10 | 9.50 | 9.50 | 11.07 |
| *Afropithecus* | 17.22 | 15.72 | 7.56 | 11.45 | 6.42 | 10.40 | 6.62 | 13.35 | 9.25 | 9.46 | 11.61 | 10.31 |
| *Cercopithecus mitis* | 8.48 | 7.48 | 5.76 | 7.04 | 4.47 | 6.70 | 4.25 | 6.77 | 6.43 | 3.74 | 5.29 | 5.85 |
|  | 0.69 | 0.77 | 1.05 | 0.69 | 0.45 | 0.61 | 0.47 | 1.01 | 0.48 | 0.47 | 0.65 | 0.52 |
| *Chlorocebus aethiops* | 6.64 | 6.13 | 3.95 | 5.02 | 3.57 | 5.33 | 3.43 | 5.85 | 5.03 | 3.35 | 4.14 | 4.63 |
|  | 0.13 | 0.47 | 0.16 | 0.57 | 0.38 | 0.27 | 0.32 | 0.59 | 0.35 | 0.42 | 0.52 | 0.24 |
| *Gorilla beringei* | 20.62 | 21.44 | 16.49 | 15.61 | 10.20 | 17.89 | 11.30 | 19.75 | 16.25 | 12.36 | 10.86 | 15.19 |
|  | 2.26 | 3.11 | 2.21 | 1.97 | 1.48 | 2.07 | 1.68 | 2.74 | 1.48 | 1.19 | 0.93 | 1.61 |
| *G. gorilla gorilla* | 22.29 | 24.49 | 17.68 | 17.92 | 10.51 | 20.04 | 12.97 | 22.57 | 16.76 | 12.64 | 11.55 | 16.54 |
|  | 3.41 | 3.37 | 2.54 | 2.44 | 2.10 | 2.66 | 1.69 | 3.19 | 2.56 | 1.97 | 2.20 | 2.20 |
| *G. gorilla graueri* | 21.67 | 22.67 | 18.01 | 15.96 | 9.64 | 18.26 | 13.27 | 19.23 | 17.03 | 11.68 | 12.27 | 15.79 |
|  | 3.16 | 3.26 | 1.66 | 1.57 | 0.77 | 1.71 | 1.67 | 2.46 | 2.22 | 1.33 | 1.99 | 1.61 |
| *Homo sapiens* | 16.21 | 17.08 | 12.62 | 11.89 | 6.85 | 14.12 | 10.15 | 15.38 | 13.79 | 9.33 | 9.12 | 11.95 |
|  | 1.94 | 1.68 | 1.54 | 1.86 | 1.23 | 1.44 | 1.43 | 1.78 | 1.68 | 0.97 | 1.16 | 1.22 |
| *Homo neanderthalensis* | 15.23 | 17.40 | 14.55 | 12.00 | 5.90 | 14.21 | 12.77 | 16.88 | 15.93 | 9.59 | 8.64 | 12.42 |
|  | 0.33 | 0.57 | 0.01 | 0.85 | 1.13 | 0.21 | 0.49 | 0.31 | 0.11 | 0.69 | 0.02 | 0.23 |
| *Hylobates lar* | 9.28 | 8.77 | 6.34 | 7.70 | 7.46 | 6.96 | 5.51 | 6.82 | 5.51 | 4.12 | 3.60 | 6.28 |
|  | 0.63 | 0.47 | 0.80 | 0.62 | 1.02 | 0.43 | 0.63 | 0.76 | 0.79 | 0.55 | 0.49 | 0.38 |
| *Lagothrix lagotricha* | 8.49 | 7.38 | 5.25 | 5.86 | 4.41 | 6.44 | 3.69 | 5.95 | 5.06 | 3.31 | 4.21 | 5.22 |
|  | 0.73 | 0.59 | 0.82 | 0.29 | 1.18 | 0.31 | 0.66 | 0.80 | 0.64 | 0.61 | 0.35 | 0.36 |
| *Macaca fascicularis* | 7.50 | 6.51 | 4.39 | 6.16 | 3.51 | 5.44 | 3.63 | 6.15 | 5.11 | 3.35 | 4.14 | 4.89 |
|  | 0.50 | 0.49 | 0.47 | 0.32 | 0.62 | 0.32 | 0.33 | 0.35 | 0.57 | 0.45 | 0.95 | 0.40 |
| *Macaca mulatta* | 9.96 | 9.47 | 5.96 | 8.54 | 6.83 | 7.63 | 4.82 | 9.02 | 6.28 | 4.94 | 5.62 | 6.95 |
|  | 0.85 | 0.76 | 0.71 | 0.76 | 0.95 | 0.81 | 0.35 | 0.87 | 1.12 | 0.73 | 0.95 | 0.59 |
| *Pan paniscus* | 14.94 | 17.32 | 12.49 | 11.25 | 6.01 | 14.03 | 9.21 | 16.24 | 12.33 | 9.48 | 8.64 | 11.46 |
|  | 0.74 | 0.99 | 0.93 | 1.32 | 1.11 | 1.20 | 0.70 | 1.30 | 1.10 | 0.86 | 0.94 | 0.69 |
| *P. troglodytes schweinfurthii* | 16.01 | 17.52 | 13.64 | 11.33 | 5.61 | 14.66 | 9.35 | 15.54 | 13.06 | 9.40 | 8.70 | 11.64 |
|  | 1.61 | 1.32 | 0.86 | 1.72 | 0.93 | 1.13 | 1.32 | 1.71 | 1.05 | 1.02 | 1.09 | 0.86 |
| *P. troglodytes troglodytes* | 17.04 | 18.65 | 14.02 | 11.90 | 6.95 | 15.29 | 9.98 | 17.07 | 13.37 | 9.75 | 8.99 | 12.41 |
|  | 1.85 | 1.71 | 1.79 | 1.84 | 1.18 | 1.23 | 1.41 | 1.73 | 1.81 | 1.14 | 0.97 | 1.04 |
| *P. troglodytes verus* | 16.32 | 19.00 | 14.06 | 13.33 | 6.87 | 14.69 | 9.13 | 17.59 | 13.50 | 11.03 | 10.10 | 12.71 |
|  | 1.24 | 1.66 | 1.67 | 1.43 | 0.26 | 1.22 | 0.66 | 1.69 | 1.58 | 0.85 | 0.66 | 0.87 |
| *Papio anubis* | 14.90 | 13.10 | 7.53 | 12.25 | 6.94 | 9.75 | 8.20 | 12.15 | 11.88 | 7.76 | 8.87 | 9.92 |
|  | 1.52 | 1.78 | 0.47 | 1.04 | 1.03 | 2.53 | 1.61 | 1.49 | 1.71 | 1.31 | 0.96 | 0.91 |
| *Pongo abelii* | 19.99 | 18.86 | 15.27 | 13.80 | 7.98 | 14.27 | 9.21 | 16.80 | 16.90 | 9.18 | 9.07 | 13.06 |
|  | 2.18 | 2.38 | 2.54 | 2.36 | 1.52 | 1.52 | 0.98 | 1.78 | 1.42 | 1.33 | 1.25 | 1.28 |
| *Pongo pygmaeus* | 20.53 | 19.82 | 15.72 | 15.40 | 7.96 | 15.01 | 10.70 | 17.92 | 16.41 | 9.52 | 9.26 | 13.67 |
|  | 3.11 | 2.32 | 2.12 | 2.05 | 1.16 | 2.14 | 2.25 | 2.39 | 2.65 | 1.44 | 1.71 | 1.65 |
| *Presbytis* | 8.11 | 7.73 | 5.48 | 7.31 | 4.74 | 6.86 | 4.37 | 7.03 | 5.39 | 4.52 | 5.26 | 5.92 |
|  | 0.64 | 0.25 | 0.05 | 0.16 | 0.46 | 0.71 | 0.18 | 0.59 | 0.83 | 0.24 | 0.14 | 0.12 |
| *Proconsul heseloni* | 11.10 | 9.81 | 6.17 | 8.59 | 5.75 | 7.71 | 4.51 | 9.24 | 5.52 | 5.81 | 6.67 | 7.07 |
|  | 1.09 | 0.11 | 0.64 | 0.12 | 1.00 | 0.69 | 0.40 | 0.45 | 0.21 | 0.56 | 0.81 | 0.14 |
| *Proconsul nyanzae* | 16.72 | 16.00 | 9.52 | 12.85 | 5.26 | 10.95 | 7.87 | 15.01 | 6.95 | 8.81 | 11.46 | 10.41 |
| *Symphalangus syndactylus* | 11.32 | 9.50 | 7.82 | 8.64 | 4.78 | 8.17 | 7.14 | 7.74 | 6.62 | 5.37 | 3.37 | 6.94 |
|  | 0.87 | 0.87 | 1.20 | 0.75 | 0.53 | 0.42 | 1.26 | 1.01 | 1.04 | 0.56 | 0.52 | 0.49 |

**(c) Triquetrum**

| **Taxon** | **BTB** | **HTB** | **LTB** | **HTLF** | **LTLF** | **BTHF** | **HTHF** | **GEOMEAN** |
| --- | --- | --- | --- | --- | --- | --- | --- | --- |
| *Alouatta* | 6.90 | 8.35 | 5.62 | 4.21 | 4.11 | 6.55 | 5.75 | 5.74 |
|  | 0.72 | 1.13 | 0.40 | 0.41 | 0.63 | 0.44 | 0.60 | 0.32 |
| *Ateles* | 8.64 | 10.11 | 7.86 | 4.11 | 4.13 | 7.08 | 6.03 | 6.47 |
|  | 0.89 | 0.71 | 1.85 | 0.40 | 0.57 | 1.25 | 0.77 | 0.46 |
| *Au. sediba* | 14.05 | 12.04 | 8.35 | 8.81 | 7.42 | 14.39 | 11.79 | 10.66 |
| *Cercopithecus mitis* | 8.41 | 8.85 | 6.26 | 4.07 | 4.79 | 6.53 | 5.10 | 6.06 |
|  | 0.64 | 0.84 | 0.48 | 0.47 | 0.42 | 0.38 | 0.50 | 0.39 |
| *Chlorocebus aethiops* | 7.51 | 7.03 | 5.26 | 3.88 | 4.48 | 6.09 | 4.47 | 5.37 |
|  | 0.86 | 0.56 | 1.10 | 0.35 | 0.93 | 1.07 | 0.78 | 0.72 |
| *Erythrocebus patas* | 7.74 | 8.83 | 6.51 | 4.73 | 5.64 | 6.20 | 5.19 | 6.25 |
|  | 1.75 | 3.90 | 2.35 | 1.74 | 2.06 | 1.22 | 1.22 | 1.91 |
| *Gorilla beringei* | 17.40 | 20.87 | 13.16 | 12.31 | 11.09 | 16.04 | 13.06 | 14.49 |
|  | 2.97 | 2.54 | 1.85 | 1.54 | 1.24 | 2.72 | 1.60 | 1.62 |
| *G. g. gorilla* | 18.81 | 20.69 | 14.09 | 13.07 | 11.63 | 17.18 | 15.08 | 15.46 |
|  | 2.08 | 4.33 | 1.70 | 1.97 | 1.56 | 1.92 | 2.24 | 1.84 |
| *G. g. graueri* | 18.92 | 17.91 | 13.54 | 12.13 | 11.44 | 16.60 | 13.78 | 14.59 |
|  | 2.83 | 3.40 | 2.13 | 1.63 | 1.93 | 1.44 | 1.63 | 1.50 |
| *Homo sapiens* | 15.33 | 14.40 | 10.59 | 9.45 | 9.24 | 13.75 | 11.12 | 11.74 |
|  | 1.71 | 1.51 | 1.29 | 0.96 | 1.16 | 1.60 | 1.25 | 1.13 |
| *H. neanderthalensis* | 16.88 | 13.83 | 9.16 | 8.75 | 8.41 | 14.49 | 11.54 | 11.47 |
|  | 0.88 | 0.04 | 0.06 | 0.92 | 0.41 | 1.00 | 1.53 | 0.68 |
| *Hylobates lar* | 8.92 | 6.78 | 4.63 | 4.38 | 3.91 | 7.67 | 5.54 | 5.72 |
|  | 0.75 | 0.62 | 0.50 | 0.44 | 0.37 | 0.98 | 0.64 | 0.34 |
| *Lagothrix lagotricha* | 8.07 | 9.30 | 6.12 | 3.48 | 5.27 | 7.05 | 4.87 | 6.02 |
|  | 0.54 | 0.62 | 0.68 | 0.41 | 0.49 | 0.31 | 0.54 | 0.36 |
| *Macaca fascicularis* | 6.98 | 7.39 | 5.17 | 3.77 | 4.72 | 5.39 | 4.64 | 5.29 |
|  | 0.44 | 0.79 | 0.71 | 0.32 | 0.66 | 0.40 | 0.69 | 0.44 |
| *Macaca mulatta* | 9.33 | 10.22 | 6.92 | 4.77 | 5.72 | 7.21 | 5.75 | 6.89 |
|  | 0.64 | 0.88 | 0.65 | 0.72 | 0.72 | 0.98 | 0.72 | 0.61 |
| *Pan paniscus* | 13.72 | 11.88 | 10.10 | 8.77 | 8.60 | 12.59 | 9.94 | 10.62 |
|  | 0.73 | 0.98 | 1.35 | 0.84 | 0.93 | 1.14 | 1.20 | 0.69 |
| *P. troglodytes schweinfurthii* | 13.87 | 12.58 | 9.12 | 9.27 | 7.32 | 11.99 | 9.97 | 10.35 |
|  | 1.61 | 1.21 | 1.10 | 1.17 | 1.01 | 0.91 | 0.39 | 0.89 |
| *P. troglodytes troglodytes* | 14.16 | 14.40 | 10.32 | 10.14 | 9.05 | 13.30 | 10.77 | 11.53 |
|  | 1.30 | 1.90 | 1.66 | 1.18 | 1.15 | 1.29 | 1.25 | 1.05 |
| *P. troglodytes verus* | 14.40 | 15.63 | 10.52 | 10.70 | 9.73 | 13.57 | 11.32 | 12.09 |
|  | 0.93 | 0.86 | 0.66 | 0.86 | 0.46 | 0.59 | 0.96 | 0.58 |
| *Papio anubis* | 13.46 | 14.51 | 10.29 | 6.18 | 9.28 | 11.67 | 8.71 | 10.21 |
|  | 1.67 | 1.94 | 1.49 | 0.78 | 1.03 | 1.62 | 1.10 | 1.17 |
| *Pongo abelii* | 18.35 | 10.80 | 8.31 | 8.23 | 7.74 | 15.69 | 9.12 | 10.56 |
|  | 2.37 | 1.54 | 0.95 | 1.31 | 0.91 | 1.77 | 1.11 | 1.00 |
| *Pongo pygmaeus* | 19.41 | 11.75 | 9.20 | 9.58 | 8.51 | 16.99 | 10.17 | 11.63 |
|  | 2.52 | 1.84 | 1.52 | 1.49 | 1.42 | 1.84 | 1.71 | 1.48 |
| *Presbytis* | 8.21 | 8.61 | 5.85 | 4.22 | 4.74 | 6.87 | 4.93 | 5.98 |
|  | 0.45 | 0.40 | 0.02 | 0.04 | 1.29 | 0.77 | 0.16 | 0.36 |
| *Proconsul nyanzae* | 13.30 | 16.58 | 9.47 | 7.05 | 7.68 | 13.41 | 10.05 | 10.62 |
| *Symphalangus syndactylus* | 8.28 | 7.79 | 5.23 | 5.45 | 3.51 | 7.81 | 6.63 | 6.13 |
|  | 0.41 | 0.42 | 0.50 | 0.14 | 0.89 | 0.44 | 0.67 | 0.26 |
| *Theropithecus gelada* | 11.17 | 12.07 | 8.82 | 5.12 | 6.58 | 9.79 | 6.77 | 8.25 |
|  | 0.62 | 0.78 | 0.89 | 0.36 | 0.78 | 0.97 | 0.73 | 0.29 |

**(d) Capitate**

| **Taxon** | **LCB** | **HCB** | **BCB** | **BCPF** | **HCPF** | **BCN** | **GEOMEAN** |
| --- | --- | --- | --- | --- | --- | --- | --- |
| *Afropithecus* | 16.70 | 15.00 | 10.90 | 6.90 | 7.50 | 6.30 | 9.81 |
| *Alouatta palliata* | 8.32 | 6.67 | 6.98 | 3.61 | 4.32 | 3.59 | 5.27 |
|  | 0.65 | 0.65 | 0.54 | 0.40 | 0.48 | 0.32 | 0.40 |
| *Ateles* | 10.27 | 7.61 | 7.14 | 4.52 | 4.57 | 4.03 | 5.98 |
|  | 1.58 | 0.51 | 0.38 | 0.84 | 0.27 | 0.26 | 0.40 |
| *Au. afarensis* | 19.86 | 18.71 | 14.41 | 10.92 | 10.52 | 9.77 | 13.48 |
| *cf. Australopithecus* | 20.67 | 15.62 | 10.56 | 11.48 | 11.43 | 7.57 | 12.25 |
| *Au. africanus* | 18.16 | 15.82 | 13.05 | 10.29 | 11.41 | 9.10 | 12.60 |
| *Au. sediba* | 17.70 | 15.60 | 12.55 | 9.96 | 8.70 | 9.63 | 11.93 |
|  | 0.03 | 0.78 | 0.42 | 0.20 | 0.07 | 0.23 | 0.14 |
| *Cercopithecus mitis* | 10.01 | 8.08 | 7.15 | 4.25 | 5.20 | 3.80 | 6.03 |
|  | 0.93 | 0.63 | 1.18 | 0.58 | 0.61 | 0.51 | 0.67 |
| *Chlorocebus aethiops* | 8.06 | 6.76 | 5.81 | 3.82 | 4.44 | 3.04 | 5.03 |
|  | 0.42 | 0.67 | 0.41 | 0.42 | 0.37 | 0.02 | 0.26 |
| *Erythrocebus patas* | 10.32 | 8.82 | 7.44 | 4.14 | 5.53 | 3.73 | 6.21 |
|  | 1.92 | 1.89 | 1.55 | 0.69 | 0.88 | 0.38 | 1.05 |
| *Gorilla beringei* | 25.54 | 25.03 | 16.12 | 13.29 | 16.20 | 10.68 | 16.92 |
|  | 2.74 | 2.58 | 1.98 | 2.28 | 2.09 | 1.20 | 1.92 |
| *G. g. gorilla* | 28.87 | 28.70 | 18.47 | 15.70 | 18.06 | 11.95 | 19.27 |
|  | 3.98 | 3.96 | 2.94 | 2.33 | 2.86 | 1.70 | 2.54 |
| *G. g. graueri* | 27.86 | 27.42 | 16.80 | 16.07 | 16.94 | 10.88 | 18.32 |
|  | 2.70 | 2.78 | 2.13 | 1.66 | 1.76 | 1.14 | 1.67 |
| *Homo sapiens* | 22.35 | 19.01 | 14.58 | 12.05 | 12.71 | 11.80 | 14.92 |
|  | 2.06 | 1.83 | 1.85 | 1.23 | 1.47 | 1.62 | 1.35 |
| *Homo neanderthalensis* | 23.80 | 21.56 | 17.30 | 12.00 | 14.30 | 10.50 | 15.87 |
| *Hylobates lar* | 11.04 | 8.75 | 6.89 | 3.73 | 5.18 | 3.41 | 5.93 |
|  | 0.75 | 0.59 | 0.78 | 0.36 | 0.51 | 0.40 | 0.42 |
| *Lagothrix lagotricha* | 8.59 | 6.50 | 5.93 | 4.32 | 4.67 | 3.75 | 5.41 |
|  | 0.59 | 0.29 | 0.34 | 0.41 | 0.42 | 0.33 | 0.36 |
| *Macaca fascicularis* | 8.21 | 6.70 | 5.59 | 3.68 | 4.52 | 3.20 | 5.03 |
|  | 0.19 | 0.44 | 0.72 | 0.22 | 0.50 | 0.36 | 0.29 |
| *Macaca mulatta* | 11.05 | 9.42 | 8.05 | 4.96 | 5.87 | 4.05 | 6.79 |
|  | 0.72 | 1.12 | 0.76 | 0.49 | 0.60 | 0.45 | 0.59 |
| *Pan paniscus* | 22.68 | 17.61 | 14.21 | 10.99 | 13.25 | 8.17 | 13.73 |
|  | 0.93 | 0.98 | 0.97 | 0.60 | 1.24 | 0.89 | 0.68 |
| *P. troglodytes schweinfurthii* | 22.75 | 19.56 | 14.76 | 10.83 | 13.59 | 8.15 | 14.08 |
|  | 1.84 | 1.49 | 1.50 | 0.59 | 1.41 | 0.93 | 0.99 |
| *P. troglodytes troglodytes* | 23.85 | 20.93 | 15.28 | 11.69 | 14.14 | 8.68 | 14.87 |
|  | 1.94 | 1.74 | 1.33 | 0.95 | 0.90 | 1.50 | 1.13 |
| *P. troglodytes verus* | 23.84 | 20.48 | 14.68 | 11.85 | 14.24 | 9.10 | 14.89 |
|  | 1.58 | 1.48 | 1.07 | 0.81 | 1.70 | 1.00 | 0.95 |
| *Papio anubis* | 16.85 | 14.02 | 11.71 | 7.22 | 8.73 | 6.02 | 10.08 |
|  | 1.34 | 1.87 | 1.05 | 0.61 | 0.84 | 0.67 | 0.94 |
| *Pongo abelii* | 24.87 | 20.89 | 14.87 | 10.30 | 12.37 | 7.74 | 13.99 |
|  | 2.99 | 1.69 | 1.50 | 1.53 | 1.56 | 1.40 | 1.49 |
| *Pongo pygmaeus* | 24.92 | 20.93 | 16.18 | 10.05 | 12.90 | 7.99 | 14.30 |
|  | 3.04 | 2.57 | 1.91 | 1.91 | 1.71 | 1.55 | 1.75 |
| *Presbytis* | 10.23 | 7.75 | 7.34 | 4.44 | 5.30 | 4.36 | 6.24 |
|  | 0.54 | 0.38 | 0.53 | 0.29 | 0.21 | 0.37 | 0.17 |
| *Proconsul africanus* | 12.35 | 9.19 | 7.30 | 5.04 | 6.11 | 4.13 | 6.87 |
| *Proconsul heseloni* | 13.22 | 9.72 | 7.61 | 5.43 | 6.50 | 4.12 | 7.22 |
|  | 1.83 | 1.45 | 1.20 | 0.99 | 1.09 | 0.77 | 1.13 |
| *Rudapithecus* | 16.65 | 13.25 | 9.44 | 6.54 | 8.01 | 5.16 | 9.09 |
| *Sivapithecus indicus* | 21.92 | 20.65 | 12.81 | 10.45 | 11.91 | 7.78 | 13.33 |
| *Symphalangus syndactylus* | 13.15 | 9.96 | 7.98 | 4.28 | 6.39 | 4.22 | 7.02 |
|  | 0.46 | 0.48 | 0.42 | 0.22 | 0.60 | 0.15 | 0.20 |
| *Theropithecus gelada* | 13.92 | 11.04 | 9.64 | 5.94 | 7.07 | 5.00 | 8.23 |
|  | 0.84 | 0.39 | 0.61 | 0.31 | 0.26 | 0.29 | 0.33 |

**(e) Scaphoid**

| **Taxon** | **LSB** | **HSB** | **BSB** | **HSRF** | **LSRF** | **HSLF** | **LSLF** | **GEOMEAN** |
| --- | --- | --- | --- | --- | --- | --- | --- | --- |
| *Alouatta* | 11.68 | 6.80 | 4.27 | 6.88 | 7.03 | 5.06 | 5.64 | 6.44 |
|  | 0.82 | 0.39 | 0.32 | 0.36 | 0.43 | 1.04 | 0.59 | 0.44 |
| *Ateles* | 14.11 | 8.84 | 5.85 | 8.60 | 9.51 | 6.26 | 8.17 | 8.42 |
|  | 1.19 | 1.08 | 0.46 | 0.60 | 1.22 | 0.87 | 1.04 | 0.75 |
| *Au. sediba* | 21.02 | 12.19 | 9.82 | 10.74 | 13.34 | 7.41 | 8.36 | 11.22 |
| *Cebus apella* | 8.85 | 5.98 | 4.54 | 6.43 | 5.90 | 4.78 | 4.86 | 5.76 |
|  | 0.11 | 0.03 | 0.08 | 0.10 | 0.05 | 0.46 | 0.28 | 0.06 |
| *Cercopithecus mitis* | 11.95 | 7.48 | 6.75 | 7.46 | 8.47 | 5.45 | 8.59 | 7.78 |
|  | 1.28 | 0.85 | 0.79 | 0.90 | 0.85 | 1.90 | 1.07 | 0.87 |
| *Chlorocebus aethiops* | 9.79 | 5.62 | 5.93 | 6.70 | 6.17 | 4.01 | 6.53 | 6.17 |
|  | 1.49 | 0.17 | 0.45 | 0.89 | 0.54 | 1.08 | 0.74 | 0.47 |
| *Erythrocebus patas* | 12.62 | 8.65 | 7.85 | 8.26 | 9.19 | 5.45 | 6.44 | 8.00 |
|  | 0.94 | 0.69 | 1.26 | 1.22 | 1.28 | 0.21 | 2.81 | 0.43 |
| *Gorilla beringei* | 38.90 | 24.11 | 14.89 | 21.39 | 20.99 | 13.34 | 11.32 | 19.09 |
|  | 8.28 | 4.03 | 2.45 | 2.43 | 2.32 | 1.86 | 1.38 | 2.31 |
| *G. g. gorilla* | 40.65 | 24.77 | 16.17 | 23.14 | 22.73 | 15.40 | 13.36 | 20.83 |
|  | 5.75 | 4.00 | 2.81 | 3.21 | 3.85 | 2.55 | 2.70 | 2.75 |
| *G. g. graueri* | 39.32 | 23.15 | 15.15 | 22.95 | 21.15 | 15.75 | 13.96 | 20.37 |
|  | 4.95 | 2.80 | 2.74 | 3.97 | 2.82 | 2.60 | 1.85 | 2.81 |
| *Homo sapiens* | 26.60 | 15.00 | 13.45 | 14.00 | 17.16 | 9.02 | 8.36 | 13.75 |
|  | 2.93 | 2.23 | 2.19 | 2.07 | 2.49 | 1.93 | 1.86 | 1.71 |
| *Homo neanderthalensis* | 26.62 | 15.98 | 14.91 | 15.40 | 19.90 | 8.44 | 8.30 | 14.50 |
|  | 1.11 | 0.95 | 0.30 | 0.85 | 0.14 | 0.37 | 1.27 | 0.35 |
| *Hylobates lar* | 15.81 | 8.38 | 6.38 | 7.29 | 8.87 | 6.16 | 8.14 | 8.28 |
|  | 1.27 | 0.59 | 1.11 | 0.98 | 0.74 | 0.56 | 0.74 | 0.55 |
| *Lagothrix lagotricha* | 11.89 | 8.08 | 5.60 | 7.67 | 9.05 | 5.87 | 5.10 | 7.30 |
|  | 0.85 | 0.74 | 0.58 | 0.70 | 0.92 | 0.83 | 0.59 | 0.60 |
| *Macaca fascicularis* | 11.23 | 6.27 | 6.36 | 7.10 | 6.24 | 4.18 | 6.43 | 6.56 |
|  | 0.91 | 0.64 | 0.56 | 0.61 | 0.70 | 0.80 | 1.31 | 0.67 |
| *Macaca mulatta* | 14.23 | 8.81 | 8.82 | 9.48 | 8.53 | 4.64 | 7.52 | 8.43 |
|  | 1.52 | 0.82 | 0.93 | 1.34 | 0.69 | 0.44 | 1.78 | 0.75 |
| *Oreopithecus* | 21.76 | 13.80 | 8.45 | 13.63 | 14.10 | 8.80 | 11.00 | 12.48 |
| *Pan paniscus* | 27.86 | 18.39 | 11.26 | 16.83 | 17.17 | 10.65 | 8.66 | 14.73 |
|  | 1.71 | 1.36 | 0.89 | 1.63 | 1.41 | 0.99 | 1.21 | 0.75 |
| *P. troglodytes schweinfurthii* | 28.22 | 17.87 | 11.62 | 18.56 | 16.87 | 10.81 | 9.49 | 15.18 |
|  | 2.94 | 1.86 | 1.25 | 2.65 | 1.08 | 1.64 | 1.63 | 1.67 |
| *P. troglodytes troglodytes* | 29.94 | 18.58 | 12.46 | 18.77 | 17.90 | 10.88 | 8.51 | 15.43 |
|  | 3.02 | 2.30 | 1.81 | 2.41 | 2.15 | 1.62 | 1.93 | 1.62 |
| *P. troglodytes verus* | 28.19 | 19.07 | 12.37 | 18.52 | 18.62 | 10.00 | 10.24 | 15.68 |
|  | 2.96 | 1.60 | 1.08 | 1.55 | 1.47 | 0.86 | 1.22 | 1.16 |
| *Papio anubis* | 18.83 | 12.39 | 12.33 | 14.08 | 12.73 | 6.87 | 10.68 | 12.05 |
|  | 2.03 | 1.77 | 1.25 | 1.76 | 1.93 | 1.00 | 1.74 | 1.31 |
| *Pongo abelii* | 31.04 | 18.33 | 13.41 | 16.33 | 17.05 | 9.52 | 14.87 | 16.18 |
|  | 4.86 | 2.29 | 2.31 | 1.30 | 2.78 | 2.27 | 2.20 | 1.93 |
| *Pongo pygmaeus* | 30.58 | 16.82 | 13.22 | 16.61 | 16.24 | 9.46 | 15.72 | 15.97 |
|  | 3.17 | 2.55 | 2.47 | 1.74 | 2.10 | 2.22 | 2.45 | 2.00 |
| *Presbytis* | 11.66 | 7.32 | 6.60 | 7.58 | 8.88 | 4.46 | 9.23 | 7.67 |
|  | 0.55 | 0.17 | 0.11 | 0.28 | 0.18 | 0.23 | 0.25 | 0.02 |
| *Proconsul africanus* | 16.36 | 10.33 | 5.73 | 9.62 | 10.35 | 7.70 | 10.10 | 9.60 |
| *Proconsul heseloni* | 16.33 | 10.29 | 6.45 | 9.69 | 9.88 | 6.60 | 9.90 | 9.45 |
|  | 0.22 | 0.05 | 0.71 | 1.12 | 1.18 | 0.85 | 0.99 | 0.22 |
| *Theropithecus gelada* | 15.69 | 10.95 | 10.51 | 10.62 | 11.94 | 7.26 | 12.87 | 11.13 |
|  | 0.87 | 0.29 | 0.56 | 1.02 | 0.78 | 1.34 | 0.76 | 0.72 |
