# Supplementary figures and images for: Different evolutionary pathways underlie the morphology of wrist bones in hominoids
Source: BMC Evol Biol. 2013 Oct 23;13:229. doi: 10.1186/1471-2148-13-229 (PMC4015765; doi:10.1186/1471-2148-13-229)

Scaphoid

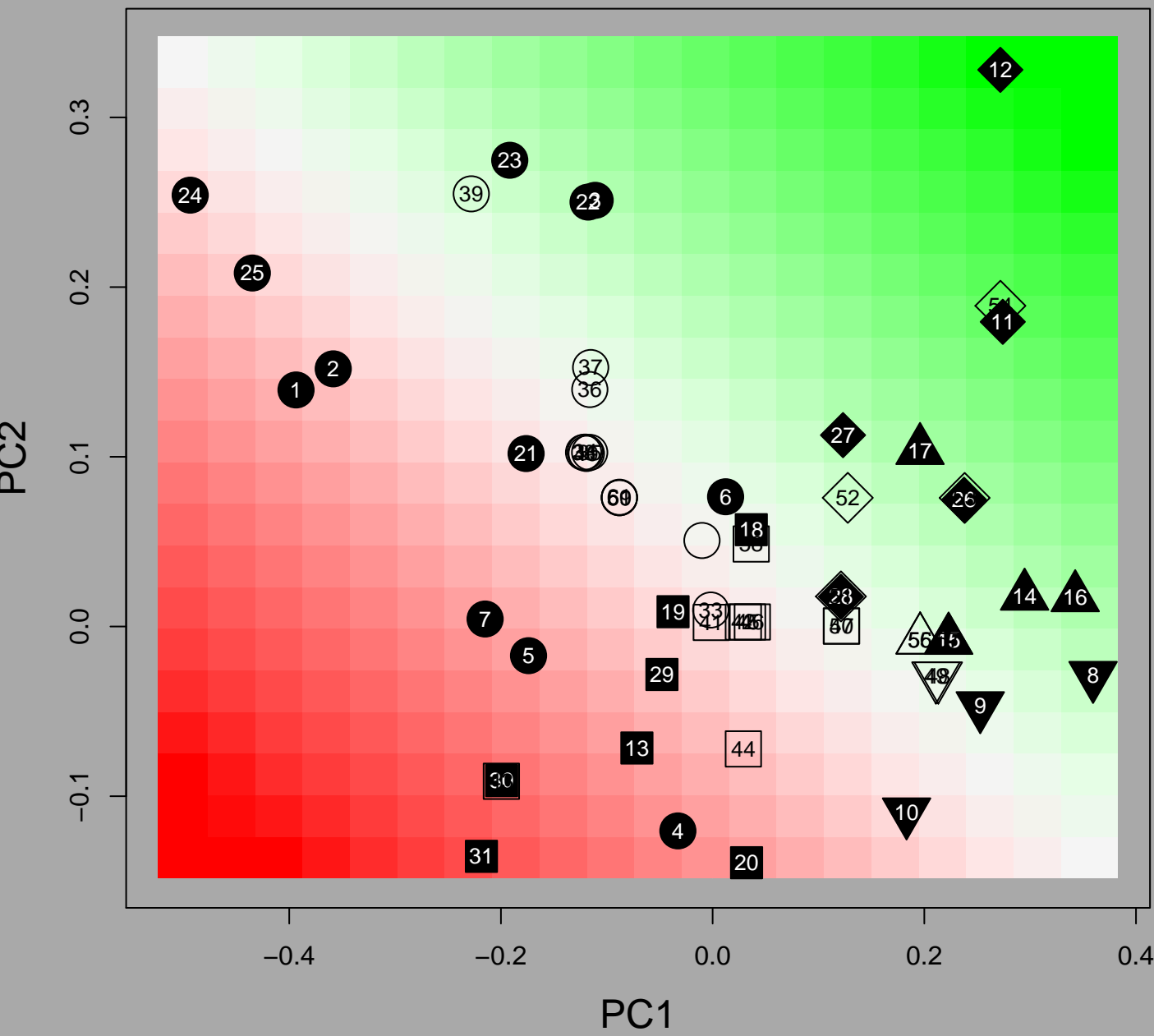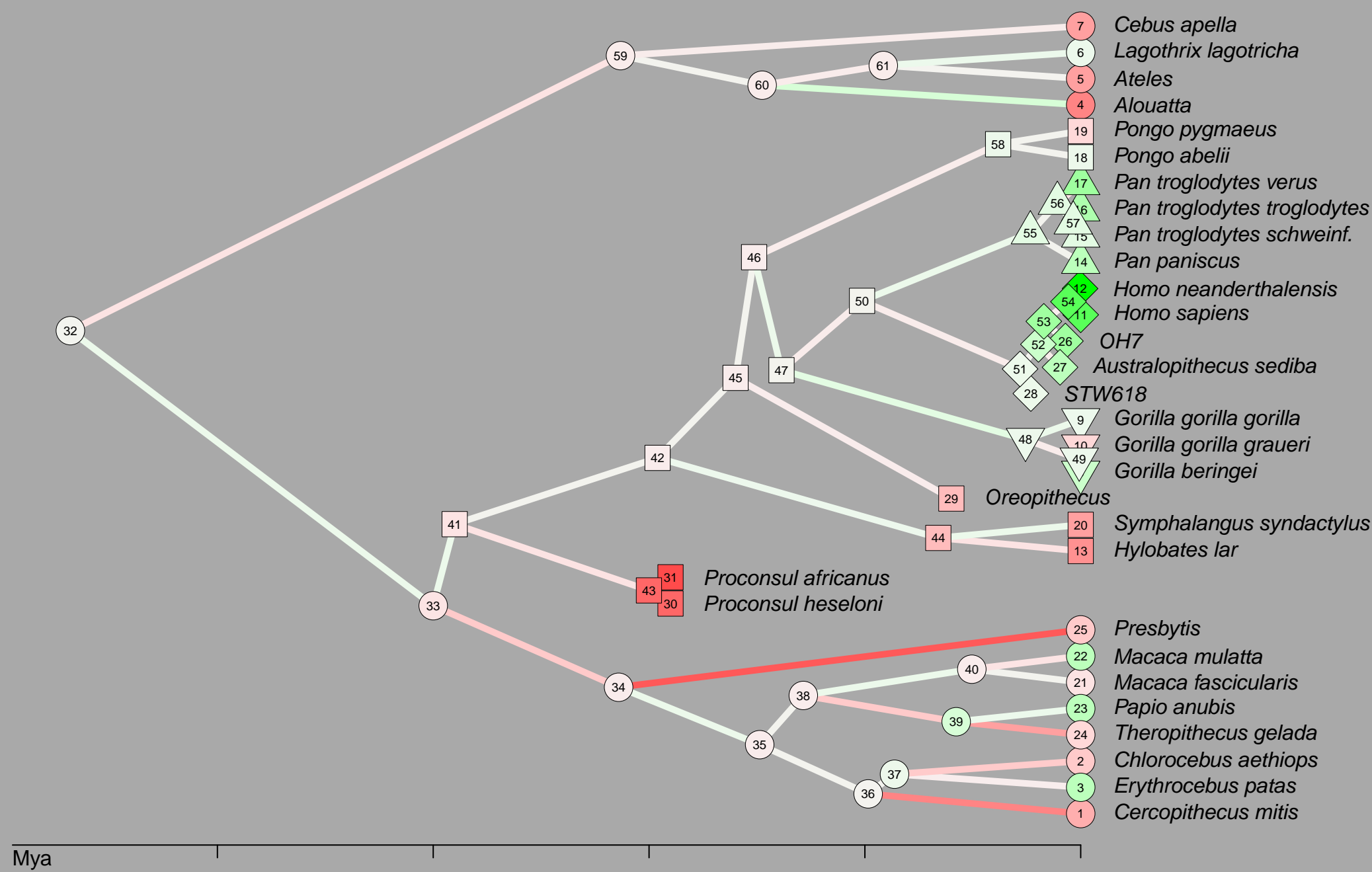

Supplement: Additional file 2 — Measurements for each wrist bone used in this analysis, shown on human bones as an example. Acronyms are the same as described in Table 3. [file 1471-2148-13-229-S2.pdf]
