# Supplementary material for: Different evolutionary pathways underlie the morphology of wrist bones in hominoids
Source: BMC Evol Biol. 2013 Oct 23;13:229. doi: 10.1186/1471-2148-13-229 (PMC4015765; doi:10.1186/1471-2148-13-229)
Supplement: Additional file 3 — Results of the phylogenetic principal component analysis (PC1 and PC2) on the scaphoid wrist variables, including all haplorrhine taxa, and the estimated ancestral states (nodes) and rates (branches) plotted in morphospace (left) and on a phylogenetic tree (right). Symbols and colour gradient the same as described in Figure 2. [file 1471-2148-13-229-S3.pdf]

Scaphoid

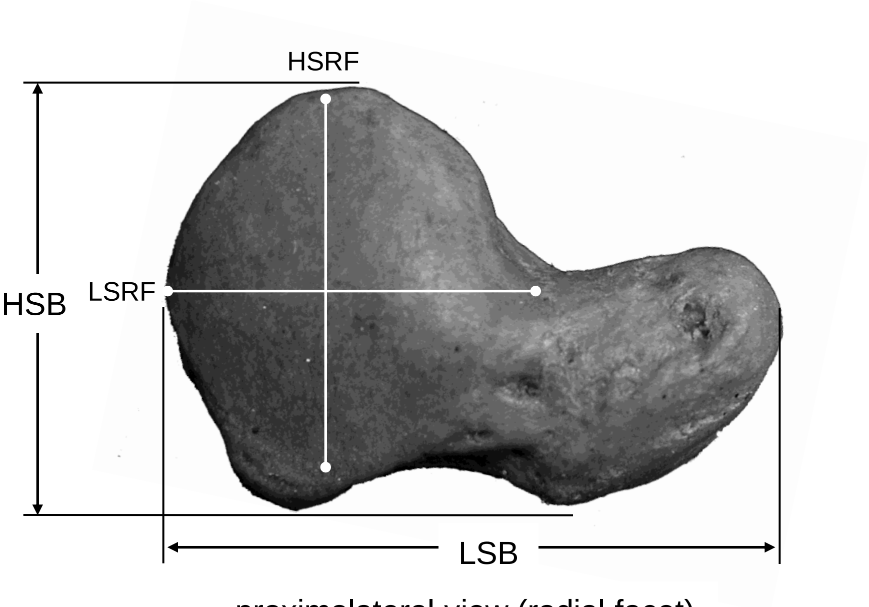

proximolateral view (radial facet)

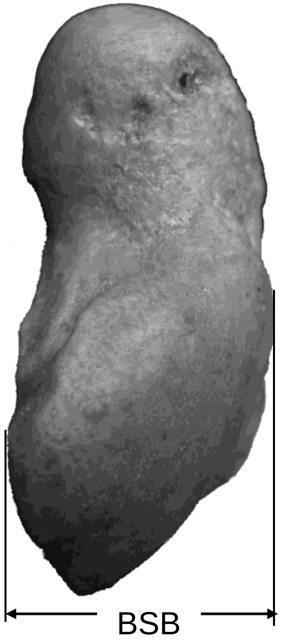

palmar view

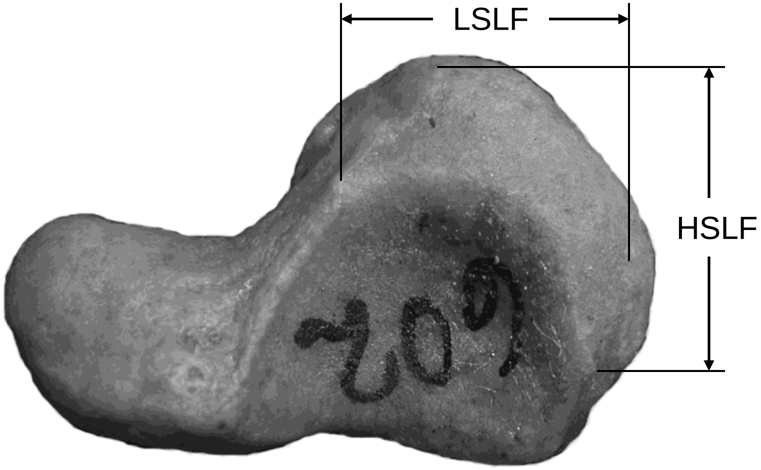

mediodistal view (capitate facet)

Lunate

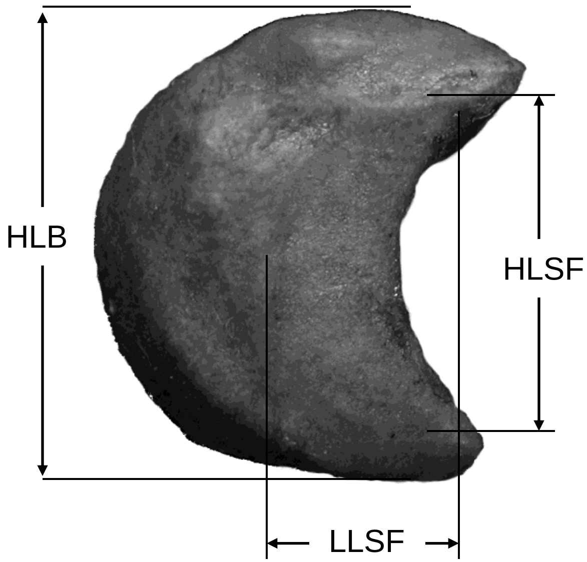

lateral view
(scaphoid facet)

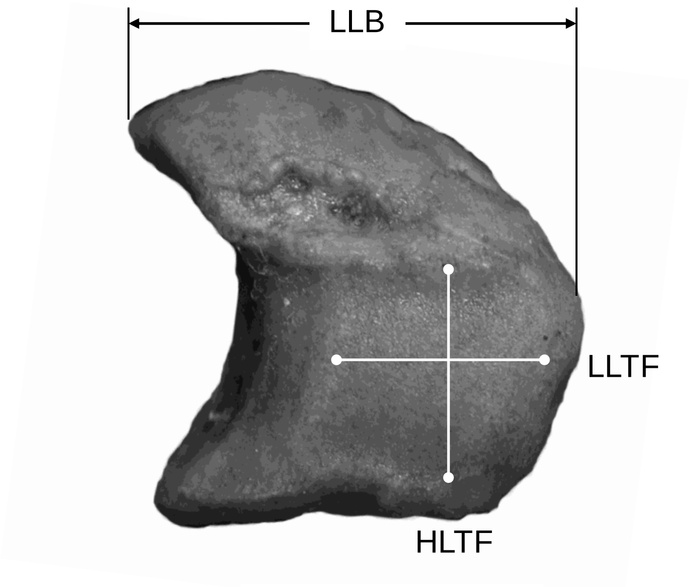

medial view
(triquetrum facet)

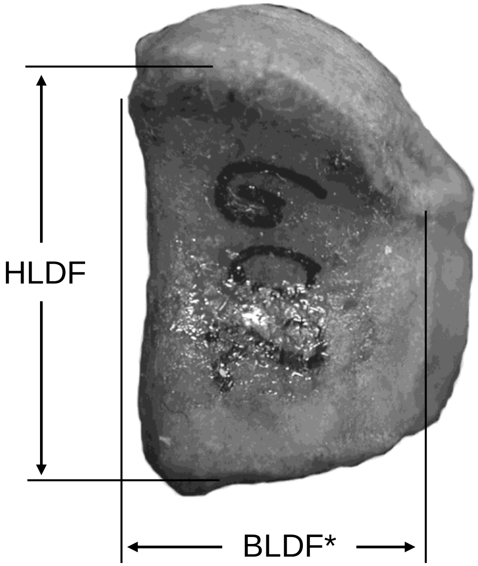

distal view
(capitate facet)

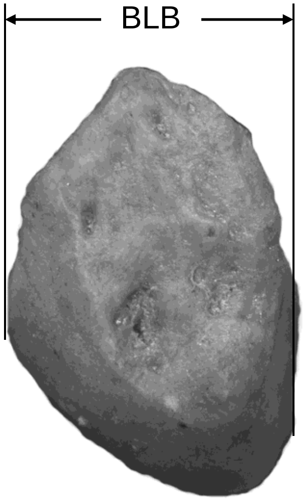

palmar view

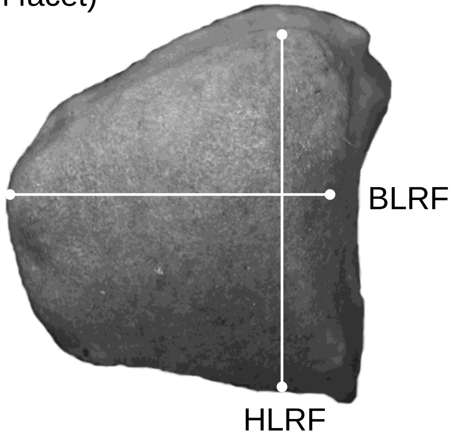

proximal view
(radial facet)

Triquetrum

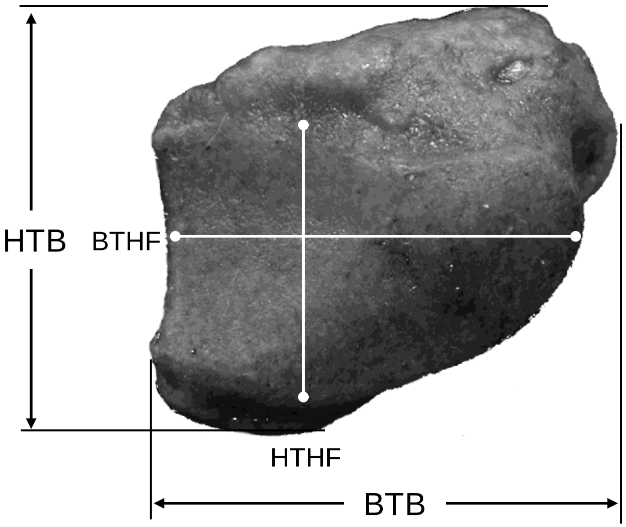

distal view
(hamate facet)

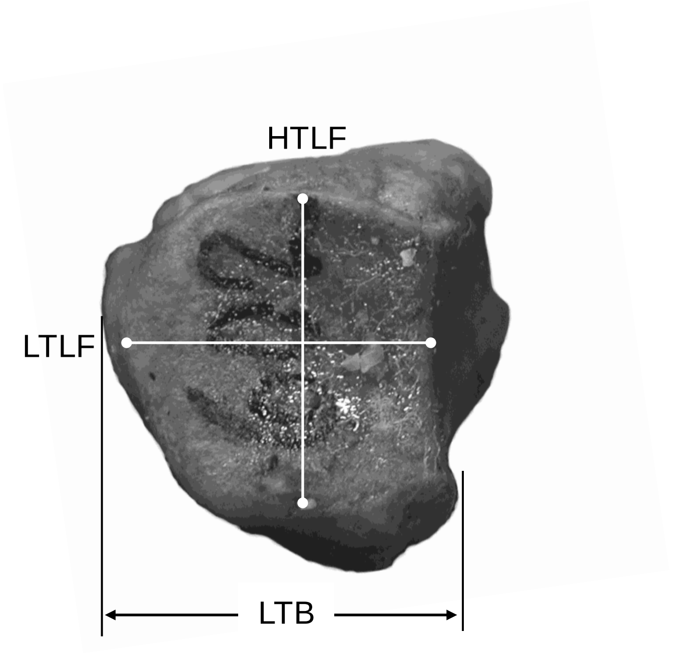

lateral view
(lunate facet)

Capitate

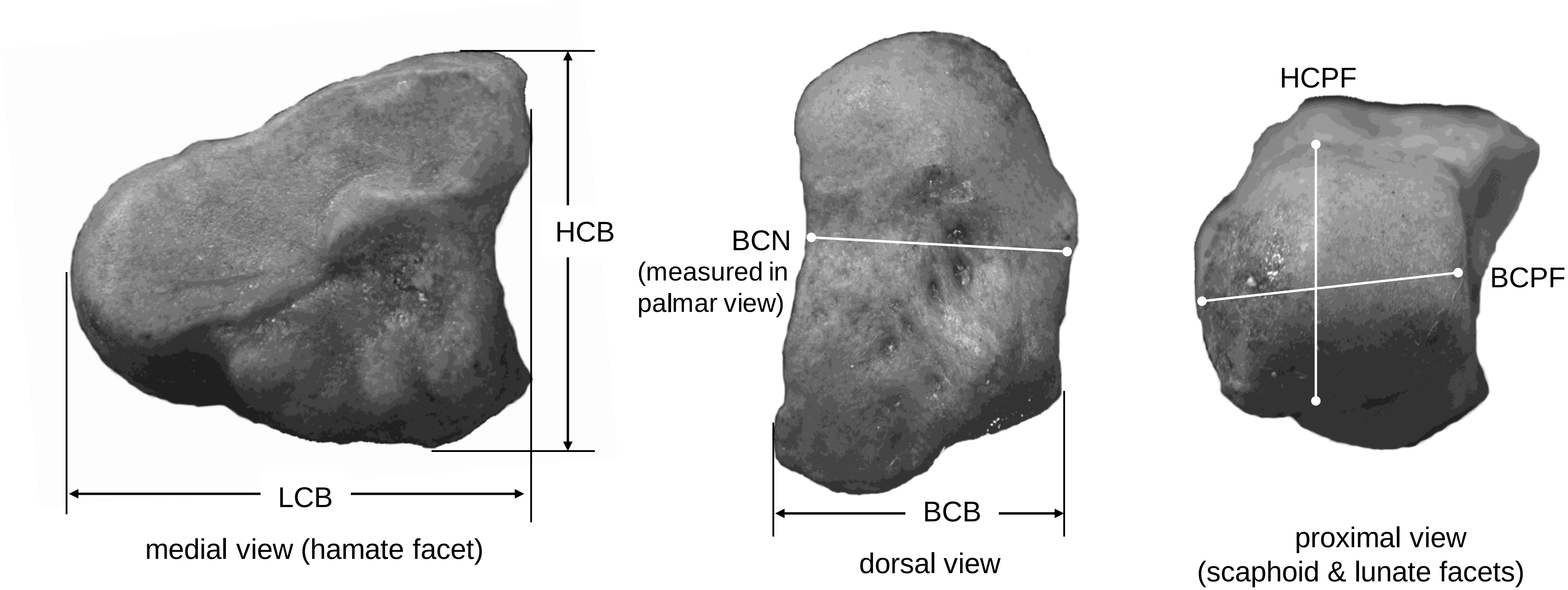
HCB
LCB
medial view (hamate facet)
BCN
(measured in
palmar view)
BCB
dorsal view
HCPF
BCPF
proximal view
(scaphoid & lunate facets)

Hamate

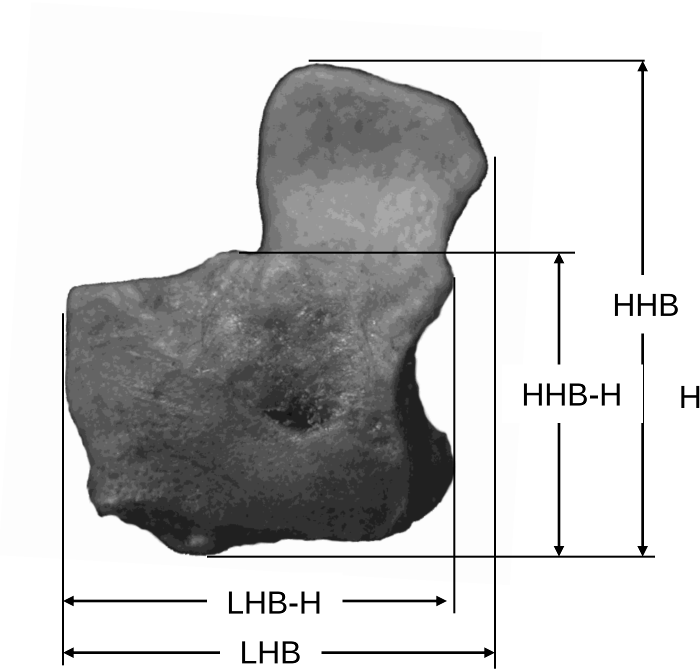

lateral view (capitate facet)

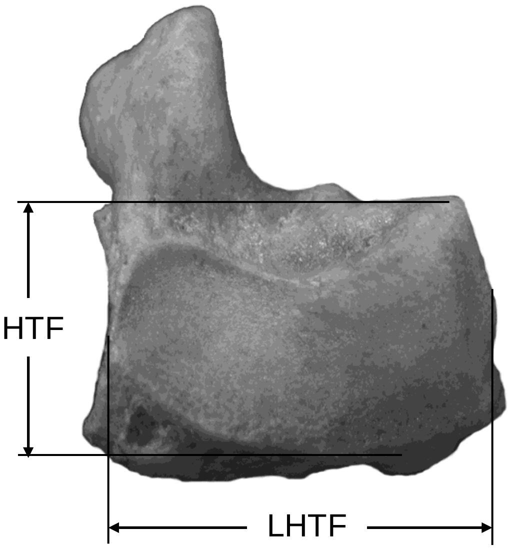

medial view (triquetrum facet)

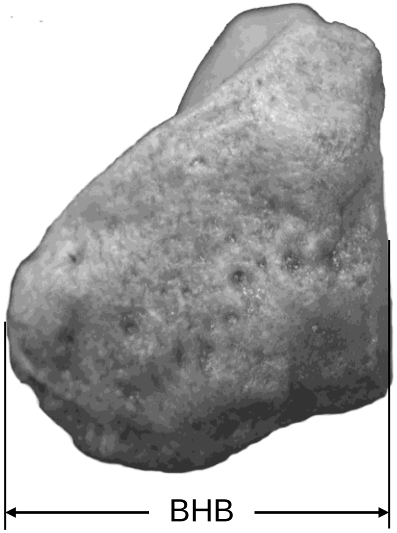

dorsal view
